# A scalable solution for multi-symptom disease prediction and lifestyle recommendations using machine learning

**DOI:** 10.1038/s41598-026-58276-6

**Published:** 2026-06-20

**Authors:** Akbar Hussain, Saif Al-jumaili, Faiza Tahir, Sehrish Munawar Cheema, Eshmal Malik, Amna Anwar, Frederico Branco, Ivan Miguel Pires

**Affiliations:** 1 Knowledge Unit of Systems and Technology, University of Management and Technology, Sialkot, Pakistan; 2 Institute of Biophysics and Biomedical Engineering (IBEB), Faculty of Sciences, University of Lisbon, Lisbon, Portugal; 3 AquaValor- Centro de Valorizaçao e Transferência de Tecnologia da Água, Chaves, Portugal; 4 Department of Computer Science, University of Management and Technology, Lahore, Pakistan; 5 Institute for Systems and Computer Engineering, Technology and Science (INESC TEC), Porto, Portugal; 6 School of Science and Technology, Universidade de Trás-os-Montes e Alto Douro, Vila Real, Portugal; 7 Instituto de Telecomunicações, Escola Superior de Tecnologia e Gestão de Águeda, Universidade de Aveiro, Águeda, Portugal

**Keywords:** Healthcare, Disease diagnosis, Machine Learning (ML), Prediction, Food recommendation, Computational biology and bioinformatics, Diseases, Health care, Mathematics and computing

## Abstract

Accurate prediction of diseases from multiple co-occurring symptoms remains an important challenge in intelligent healthcare systems. Machine-learning models can support early symptom-based risk screening; however, their clinical use is limited by dataset quality, lack of external validation, interpretability concerns, and the risk of overstated diagnostic claims. This study presents a mobile decision-support prototype for multi-symptom disease prediction and rule-based lifestyle recommendation. The experiments were conducted using the publicly available Kaggle Medicine Recommendation System Dataset, which contains 4,920 symptom-based records spanning 41 disease classes, along with supporting files for disease descriptions, precautions, medications, dietary suggestions, and workout recommendations. Seven supervised machine-learning models were evaluated, including Decision Tree, Random Forest, Naive Bayes, Logistic Regression, XGBoost, Support Vector Machine, and K-Nearest Neighbors. The best-performing models achieved high classification accuracy under the adopted evaluation protocol. To avoid overinterpretation, these results are reported as benchmark performance on a secondary public dataset rather than evidence of clinical diagnostic validity. The Android-Flask prototype links the predicted disease class to a lookup-based recommendation layer that retrieves disease-associated information from supporting datasets. The system should therefore be interpreted as a decision-support and educational prototype, not as a clinically validated diagnostic or prescribing tool. Future work should include external validation in independent clinical cohorts, clinician assessment of recommendations, robustness testing under missing or noisy symptom data, and broader evaluation across real-world healthcare settings.

## Introduction

Disease detection or prediction helps healthcare providers identify populations at elevated risk of developing specific diseases, an important element of the healthcare system^1,2^. For several decades, disease detection and prediction have been the sole responsibility of medical experts, who have used various methods to validate their predictions. About half a century ago, various automated tools, such as laboratory testing and real-time monitoring, emerged as breakthroughs in medical science^3–5^. Today, the availability of extensive patient records, high-resolution images, and modern diagnostic tools has enabled the development of more sophisticated and reliable tools to supplement original expert predictions and detections^6,7^. These advancements help improve patient care outcomes, healthcare experiences, early interventions, and the detection and prevention of multiple diseases^8^. Generally, the medical requirements of individuals with medical conditions cannot be adequately addressed using a single diagnostic technique. For example, medical practitioners do not rely solely on their own opinion when diagnosing a disease; laboratory examination reports also need to be correlated with diagnostic findings^3,4^.

The increasing availability of online medical information and electronic medical records has made the situation even more difficult, as a medical practitioner has to assess numerous symptoms and, in some cases, monitor them at continuous intervals^1,6^. This abundance of information carries countless symptoms and clues that are difficult to filter out and process manually. Therefore, specialized tools and techniques are required not only to handle the vast amount of data but also to infer meaningful information from it^9,10^. Nevertheless, advances in artificial intelligence (AI), especially machine learning (ML) and deep learning algorithms, are gaining momentum by delivering diagnostic results considered more reliable than traditional medical examination reports^6,7,11^, despite ongoing challenges in clinical and ethical validation. After COVID-19, people are much more concerned about their general health and well-being. Research shows that people with intrinsic motivation for self-care tend to achieve better health outcomes by maintaining their health before it systematically declines^12,13^.

Patients often struggle to understand their healthcare situation and options despite the abundance of online medical information, leading to inconsistent and inaccurate healthcare decisions^11^. Patients must synchronize nutritional decisions with medical treatments and physical activity scheduling^13^. These physical activities, including an exercise routine, require specific health requirements and personal alignment with their prescribed rehabilitation plans. These unmet patient requirements lead to various issues that negatively affect treatment outcomes, including unsuitable treatment plans, inadequate nutrition plans, unnecessary or excessive exercise regimens, and excessive spending on medications and laboratory tests^14,15^. Thus, there is a need for accessible digital decision-support tools that can organize symptom information and provide preliminary health-related guidance. The system presented in this paper should be understood as an early-stage mobile decision-support prototype rather than a clinically validated diagnostic platform. It uses supervised machine-learning models to classify disease categories from predefined symptom patterns in a secondary public dataset and connects the predicted class to a rule-based information retrieval layer. This layer retrieves disease-associated descriptions, precautions, medication-related information, diet suggestions, and workout guidance from supporting files. The proposed framework may be useful as an educational and preliminary tool for assistance, particularly in settings where access to health information is limited. However, any real-world healthcare deployment would require prospective clinical validation, clinician oversight, privacy safeguards, and alignment with recognized clinical guidelines. In light of this contribution, the study has the following two main objectives. The specific objectives of this study are as follows:

1. To evaluate multiple supervised machine-learning classifiers for symptom-based multi-class disease prediction using a publicly available secondary benchmark dataset.
2. To establish a clear and reproducible preprocessing pipeline, including missing-value handling, duplicate removal, symptom encoding, and disease-label encoding.
3. To compare the selected machine-learning models under a consistent evaluation protocol using accuracy, precision, recall, F1-score, and class-level performance indicators.
4. To integrate the selected prediction model into an Android-Flask mobile prototype for real-time symptom submission and disease prediction.
5. To connect the predicted disease class to a rule-based recommendation layer that retrieves disease-associated descriptions, precautions, medication-related information, diet suggestions, and workout guidance from supporting CSV files.
6. To discuss the methodological and clinical limitations of using a secondary symptom-based dataset for healthcare decision support.

Based on these objectives, the study addresses the following research questions:

- **RQ1:** How accurately can supervised machine-learning models classify disease categories from symptom-based input features in the selected public benchmark dataset?
- **RQ2:** Which classifier provides the best predictive performance under a consistent evaluation protocol?
- **RQ3:** How can a trained machine-learning model be integrated into an Android-Flask architecture for real-time symptom-based prediction?
- **RQ4:** How can the predicted disease class be linked to a rule-based recommendation layer that retrieves disease-associated precautions, medication-related information, diet suggestions, and workout guidance?
- **RQ5:** What are the main limitations of using a secondary symptom-based dataset for healthcare decision support, and what validation steps are required before clinical deployment?

We selected Decision Tree (DT), Random Forest (RF), Naive Bayes (NB), Logistic Regression (LR), XGBoost, Support Vector Machine (SVM), and K-Nearest Neighbors (KNN) because these classifiers represent different supervised learning strategies and have been widely applied in medical decision-support studies^10^. The dataset used in this study is a publicly available secondary dataset obtained from Kaggle. It should not be interpreted as a prospectively collected hospital cohort or as a clinically validated patient registry. In addition to the classification experiments, an Android-Flask prototype was developed to demonstrate real-time symptom submission, disease prediction, and retrieval of disease-associated supportive information. The following section reviews related work on machine-learning-based disease prediction and health recommendation systems.

The main contributions of this study are summarized as follows:

1. **Multi-model symptom-based disease prediction:** The study evaluates seven supervised machine-learning classifiers for multi-class disease prediction using symptom-based input features.
2. **Clarified dataset provenance:** The manuscript explicitly identifies the dataset as a publicly available secondary Kaggle dataset and avoids presenting it as a prospectively collected or clinically validated hospital cohort.
3. **Transparent preprocessing workflow:** The study describes the preprocessing pipeline, including missing-value treatment, duplicate removal, one-hot encoding of symptoms, and label encoding of disease classes.
4. **Consistent model evaluation:** The selected classifiers are compared under a unified evaluation setting, and the reported results are interpreted cautiously because high performance on a structured dataset does not necessarily imply clinical generalizability.
5. **Mobile prototype implementation:** The study demonstrates the integration of a trained machine-learning model with an Android-Flask architecture for real-time symptom submission and prediction.
6. **Rule-based supportive recommendation layer:** The system retrieves disease-associated descriptions, precautions, medication-related information, diet suggestions, and workout guidance from supporting CSV files.
7. **Clinical limitation awareness:** The study clearly states that the system is an early-stage decision-support prototype and requires external validation, clinician review, privacy assessment, and prospective evaluation before real-world clinical use.

## Literature review

In this section, we discuss the existing literature on applying ML algorithms to accurately detect diseases and generate recommendations. In a study, various ML models are combined to predict an individual’s risk of diabetes. The study used the John Diabetes Database and demonstrated 99

A stacked ensemble of Gradient Boosting, Gaussian Naive Bayes, DT, and RF classifiers achieved 100

Most disease prediction research focuses on a single dataset to predict a particular disease. Although this approach might be successful, it usually fails to provide complete care for patients with several health problems simultaneously. Deep learning algorithms, such as convolutional neural networks (CNNs), mentioned in the research, could surpass ML algorithms for image-based diagnosis and classification, providing faster processing times and higher classification accuracy. That said, since CNNs excel with visual data, one must exercise caution when applying them for anything other than image analysis. The aim going ahead is to create a more cohesive model capable of evaluating a patient’s risk for many diseases simultaneously. Still, there are obstacles to overcome, including data integration, model interpretability, and clinical validation-all of which are vital for real-world application^16^.

A few studies discuss the applications of ML for disease prediction in the health care and patient care processes. A pioneering study used a multi-criteria decision analysis process, using an AHPSort-based pre-filtering stage to eliminate unhealthy foods in the food recommender system^17^. Another robust study introduces a comprehensive health monitoring system that predicts hypertension, diabetes, and other minor medical conditions using patients’ personal information, activity levels, and laboratory test results. This system predicts the disease using three ML classifiers: DT, RF, and XGB. However, C4.5 demonstrated the highest prediction accuracy, up to 87% on the dataset. This prediction is further used to inform dietary and food recommendations for patients. However, clinical validation of the system remains in progress^18^. A similar study recorded cardiac activity rate, diabetic status, and body performance status of patients and predicted these diseases with accuracies of 73%, 78%, and 74%, respectively, using ML classifiers. This prediction is further used to provide customized exercise plans for both cardiac and diabetic patients, unlike most systems that provide a generic plan for all people^19^. Likewise, a study demonstrated disease prediction using ML classifiers NB, DT, and RF, with NB achieving up to 98% detection accuracy, followed by DT at up to 97%. The system not only predicts disease but also recommends appropriate medications. This shows the ability of such systems to support healthcare by providing accurate predictions and medication recommendations, thereby improving treatment planning^20^.

Recent healthcare AI studies have also demonstrated the use of computer-aided diagnosis, deep learning, and multi-class classification in medical decision-support contexts, including multisource COVID-19 diagnosis, retinopathy of prematurity stage classification, and comparative CNN-based medical image classification^21–23^.

The dataset used in the studies is another important factor in an enhanced prediction and recommendation system. A study that uses a similar dataset to the one we are using in this research evaluates three ML classifiers, which include DT, RF, and NB, to analyze 41 diseases through 95 selected symptoms. The results demonstrate that NB achieved a higher accuracy of 95.12% when applied with K-fold cross-validation^9^. Another recent study proposed an ML-based diagnostic model to predict type 2 diabetes using two clinical datasets. Their work compared 11 supervised ML models, such as RF, LG, and SVM, with rigorous data preprocessing and feature selection. RF recorded the highest accuracy (98.95%), followed by other models in terms of precision, recall, and F1-score^24^.

This brief overview of the existing literature indicates that ML classifiers are widely used in health and patient care departments. When focusing on disease prediction, ML classifiers combined with advanced feature selection techniques have achieved an impressive accuracy of up to 98%. This has increased the confidence of medical practitioners and researchers in using these models for several healthcare applications, including medical, food, and exercise recommendations. Despite this incredible progress, there are several shortcomings in this line of research as mentioned above. First, most of the datasets used in the studies predict a single disease or minor diseases. Second, the quality and compactness of the dataset are not validated in studies. Most studies rely on a single data source, whereas multi-model data collection faces numerous challenges. Third, the studies present ML classifiers but do not describe their applications in medical science or discuss the clinical validation procedures. In light of these gaps, this study uses a publicly available secondary symptom-disease dataset containing 4,920 records and 41 disease classes. The dataset is used as a benchmark for evaluating supervised machine-learning models and does not represent a prospectively validated clinical cohort. The following section describes the methodology, preprocessing pipeline, model evaluation protocol, mobile application architecture, and recommendation retrieval layer.

## Methodology

**Fig. 1:**
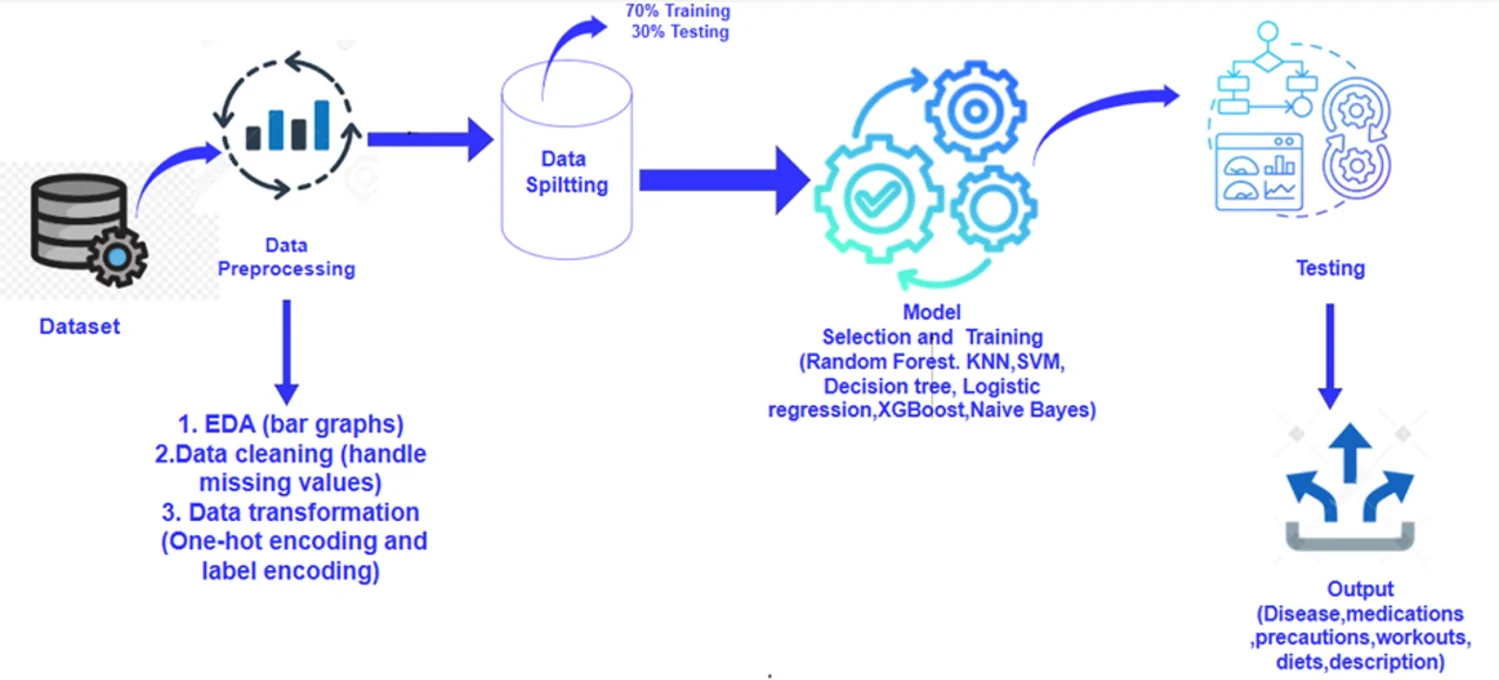
Methodology of proposed system.

The approach taken to create the disease prediction and health recommendation system is depicted in Fig. 1, which shows the entire workflow from dataset preprocessing through model training and testing to final output generation. All these processes are explained in detail in the subsequent subsections, namely, data preprocessing, data splitting, model selection and training, testing, and output generation.

### Dataset

This study used the publicly available Kaggle Medicine Recommendation System Dataset^25^. The dataset contains 4,920 symptom-based records distributed across 41 disease classes. Each record represents a symptom pattern associated with a target disease label. The input variables represent symptoms, while the target variable represents the disease class or prognosis.

The dataset was used as a secondary public benchmark dataset for machine-learning evaluation. It should not be interpreted as a prospectively collected hospital cohort or as a clinically validated patient registry. This clarification is important because public symptom-disease datasets may contain structured symptom patterns that are useful for algorithmic benchmarking but may not fully reflect the variability, uncertainty, and incomplete reporting found in real clinical environments.

In addition to the main symptom-disease dataset, several supporting CSV files were used for the recommendation layer. These files include disease descriptions, precautions, medication-related information, diet suggestions, and workout recommendations. These supporting datasets were not used as predictive input features during model training. Instead, they were queried after disease prediction to retrieve disease-associated supportive information for display in the mobile application.

Therefore, the dataset structure supports two separate components of the proposed framework: first, supervised disease classification from symptom features; and second, rule-based retrieval of disease-associated supportive information. This separation ensures that the predictive model and the recommendation layer are described accurately and avoids overstating the system as a fully personalized clinical recommendation engine.

### Data preprocessing

**Fig. 2:**
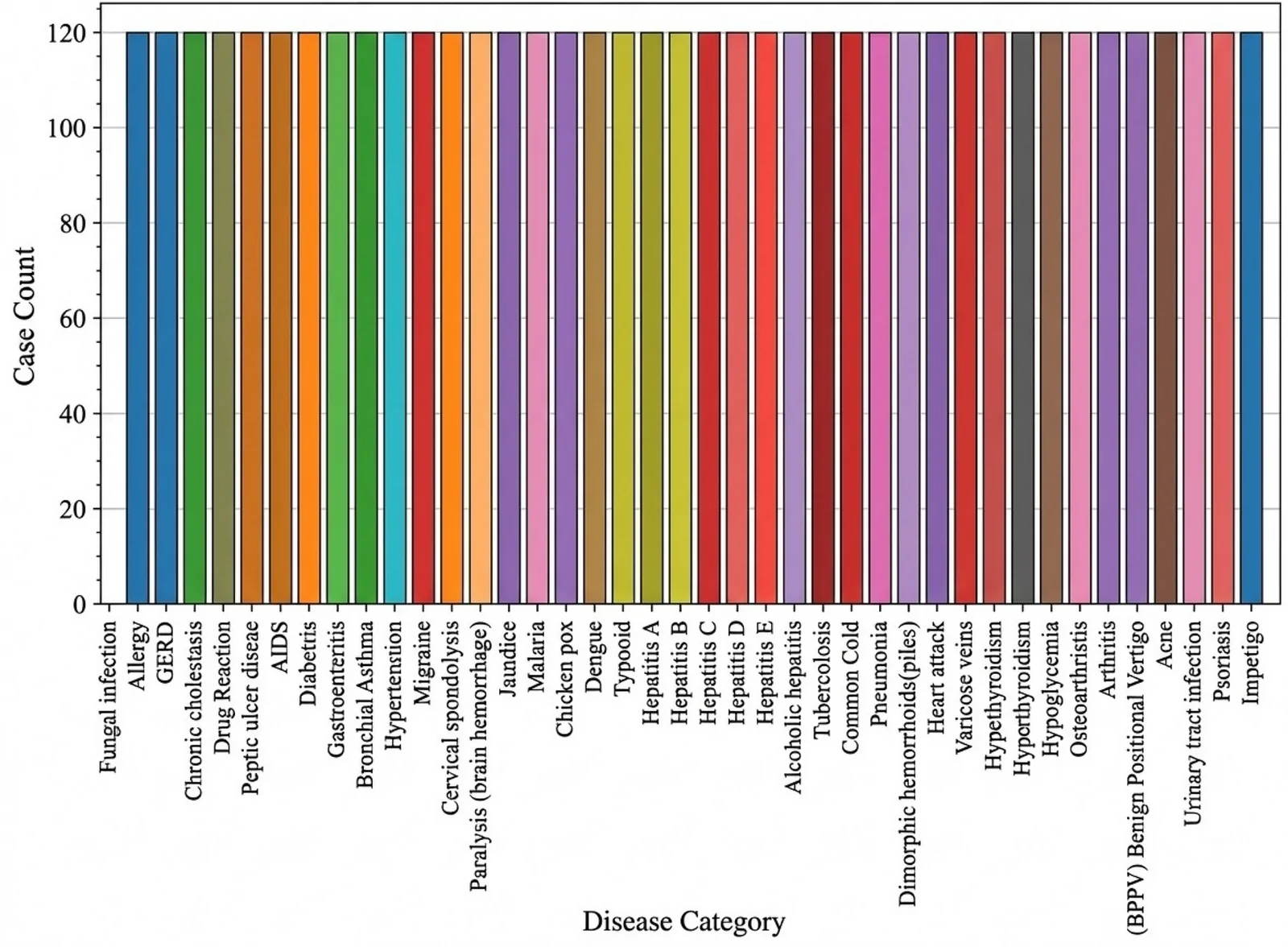
Disease distribution.

**Fig. 3:**
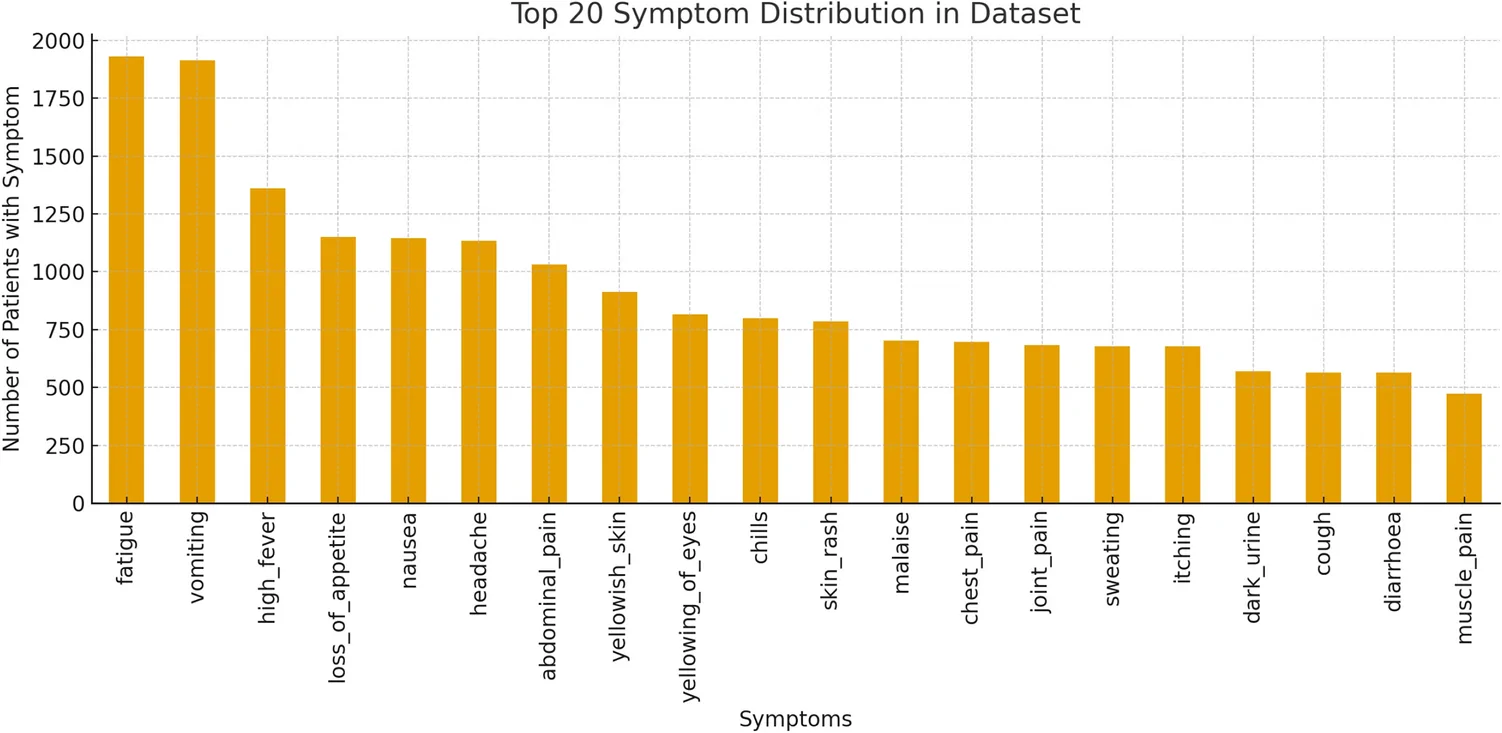
Symptoms distribution.

Data preprocessing was performed to convert the raw symptom records into a structured feature matrix suitable for supervised machine-learning classification. First, exploratory data analysis was conducted to inspect the distribution of disease classes and symptom frequencies. This step was used to identify whether the dataset contained obvious imbalance, missing entries, duplicate records, or irregular symptom values. The disease-class distribution and symptom-frequency distribution are shown in Figs. 2 and 3, respectively.

Missing values were then examined. In the raw symptom-entry file, the Symptom_4 column contained 348 missing entries. These missing values were handled using mode imputation. Under this approach, each missing value is replaced by the most frequent symptom value observed in the same column. Mode imputation was selected because the symptom fields are categorical rather than continuous numerical variables. After imputation, duplicate records were removed to reduce repeated symptom patterns and to improve the reliability of model evaluation.

The categorical symptom entries were then transformed into binary symptom indicators using one-hot encoding. In the resulting representation, each unique symptom is represented as a separate feature. A value of 1 indicates that the symptom is present in a given record, while a value of 0 indicates that the symptom is absent. This transformation converts the original symptom list into a machine-readable input vector suitable for supervised classifiers.

The target disease label was encoded using label encoding, with each disease class assigned a numerical index. The final preprocessed dataset consisted of a feature matrix *X* representing encoded symptoms and a target vector *y* representing disease labels. Because the dataset showed a balanced distribution of diseases, synthetic oversampling methods such as SMOTE were not applied. Instead, model performance was evaluated using class-level metrics, including per-class precision, recall, F1-score, and confusion-matrix analysis, to ensure that the interpretation did not rely on accuracy alone.

### Evaluation protocol

To resolve inconsistencies in the evaluation description, the final model performance was assessed using stratified 5-fold cross-validation. Stratification was used to preserve the distribution of the 41 disease classes across folds. In each iteration, four folds were used for training, and the remaining fold was used for validation. This procedure was repeated 5 times, with each fold serving once as the validation subset.

The reported performance values were calculated as the mean and standard deviation across the five validation folds. Accuracy was used as the primary metric, while precision, recall, F1-score, macro-averaged F1-score, weighted F1-score, and confusion matrix analysis were used to provide a more complete evaluation of the classifier’s behavior. This is particularly important in multi-class disease prediction because a high overall accuracy may hide class-specific errors. To further assess whether the observed differences between classifiers were statistically meaningful, pairwise statistical significance testing was performed. Because all classifiers were evaluated on the same stratified cross-validation folds, the Wilcoxon signed-rank test was used to compare paired fold-level performance scores, including accuracy and macro-F1-score. In addition, McNemar’s test was applied to paired out-of-fold predictions to assess whether the two classifiers exhibited significantly different error patterns on the same validation samples. For each model pair, the null hypothesis assumed that there was no statistically significant difference between the two classifiers. A significance level of *α = 0.05* was adopted. Because multiple pairwise comparisons were performed, Holm–Bonferroni correction was applied to control the family-wise error rate. The resulting adjusted *p*-values were reported alongside the cross-validation metrics to enable a more rigorous comparison of classifier performance.

The 70/30 train-test split was not used as the main source of the final reported results. If a 70/30 split was used during software testing or prototype development, it was treated only as an implementation check and not as the primary evaluation protocol. This distinction ensures that the methodology and results sections report one consistent evaluation pipeline.

### Model training and hyperparameter configuration

Seven supervised machine-learning classifiers were evaluated: Decision Tree, Random Forest, Naive Bayes, Logistic Regression, XGBoost, Support Vector Machine, and K-Nearest Neighbors. These models were selected because they represent different learning strategies, including tree-based classification, ensemble learning, probabilistic classification, linear classification, margin-based classification, boosting, and instance-based learning.

All models were trained on the same preprocessed feature matrix to ensure a fair comparison in the present study. Hyperparameters were selected either from commonly used default settings or tuned during training, depending on the model. The final hyperparameter settings should be reported explicitly to improve reproducibility. Table 1 summarizes the main configuration of each model.

**Table 1:** Main hyperparameter settings used for the evaluated machine-learning models.

**Model**	**Hyperparameter configuration**
Decision Tree	Criterion = Gini; maximum depth = None; minimum samples split = 2; minimum samples leaf = 1; random state = 42
Random Forest	Number of estimators = 100; criterion = Gini; maximum depth = None; minimum samples split = 2; minimum samples leaf = 1; random state = 42
Naive Bayes	Model = Multinomial Naive Bayes; smoothing parameter *α* = 1.0
Logistic Regression	Penalty = L2; solver = lbfgs; maximum iterations = 1000; regularization strength *C* = 1.0; multi-class strategy = auto
XGBoost	Number of estimators = 100; learning rate = 0.10; maximum depth = 3; subsample = 1.0; column sample by tree = 1.0; objective = multi-class classification; evaluation metric = multi-class log-loss
Support Vector Machine	Kernel = radial basis function; regularization parameter *C* = 1.0; *γ* = scale; decision function shape = one-vs-one
K-Nearest Neighbors	Number of neighbors *k* = 5; distance metric = Euclidean; weighting = uniform

### System architecture

The proposed framework consists of four main components: the data preprocessing module, the machine-learning prediction module, the Flask API layer, and the Android mobile interface. The preprocessing module converts raw symptom entries into binary feature vectors. The machine-learning prediction module uses the trained classifier to estimate the most likely disease class from the submitted symptom vector. The Flask API acts as an intermediate communication layer between the trained model and the Android application. Finally, the Android interface allows users to submit symptoms and view the predicted disease, along with supporting disease-related information.

**Fig. 4:**
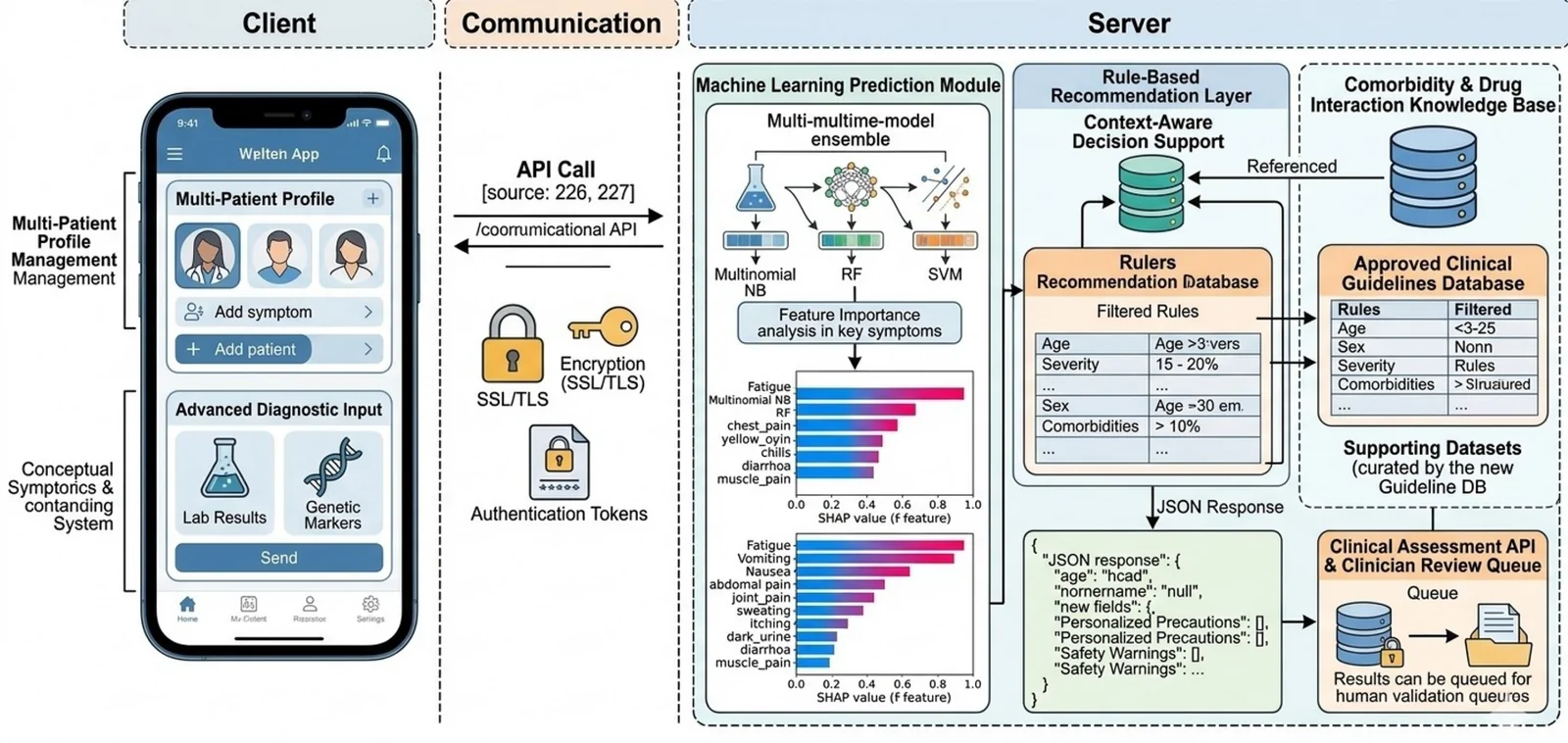
System architecture of the proposed mobile decision-support prototype. User-entered symptoms are submitted via the Android interface and forwarded to the Flask API, where they are converted into the trained binary feature representation. The machine-learning model predicts the most likely disease class, which is then linked to a rule-based recommendation layer that retrieves disease-associated descriptions, precautions, medication-related information, diet suggestions, and workout guidance from supporting files.

Figure 4 illustrates the complete data flow from symptom entry to prediction and recommendation retrieval. The architecture separates disease prediction from recommendation retrieval. This separation is important because the prediction model performs supervised classification, while the recommendation layer retrieves predefined disease-associated information from supporting files.

## Results and discussion

This section presents the experimental evaluation of our Disease Prediction and Health Recommendation System, which was developed as a mobile application. The experimentation process will involve two significant parts: (i) Android application design, and (ii) machine learning algorithms to predict diseases and provide recommendations.

### Android-flask integration for disease prediction

A Python Flask back end was implemented to enable real-time disease prediction via a /predict API endpoint. The Android application submits user-selected symptoms to the Flask server using POST requests. On the server side, the submitted symptoms are standardized and converted into the same binary feature representation used during model training. This step is necessary because the deployed model expects an input vector with the same feature order and dimensionality as the training data. The deployed prototype used the Multinomial Naive Bayes model because it provided high classification performance while remaining computationally lightweight for real-time inference. This model was therefore selected for the mobile demonstration layer. However, the experimental comparison still reports the performance of all evaluated classifiers to provide a broader assessment of model behavior.

After the disease class is predicted, the predicted label is passed to the recommendation retrieval layer. This layer queries supporting CSV files to retrieve disease-associated descriptions, precautions, medication-related information, diet suggestions, and workout recommendations. The final response is returned to the Android application in JSON format and displayed to the user. This integration demonstrates the feasibility of connecting a trained machine-learning classifier to a mobile health interface. Nevertheless, the system should be interpreted as a prototype for decision support and health information retrieval, not as a clinically validated diagnostic or prescribing platform.

### User interface development

**Fig. 5:**
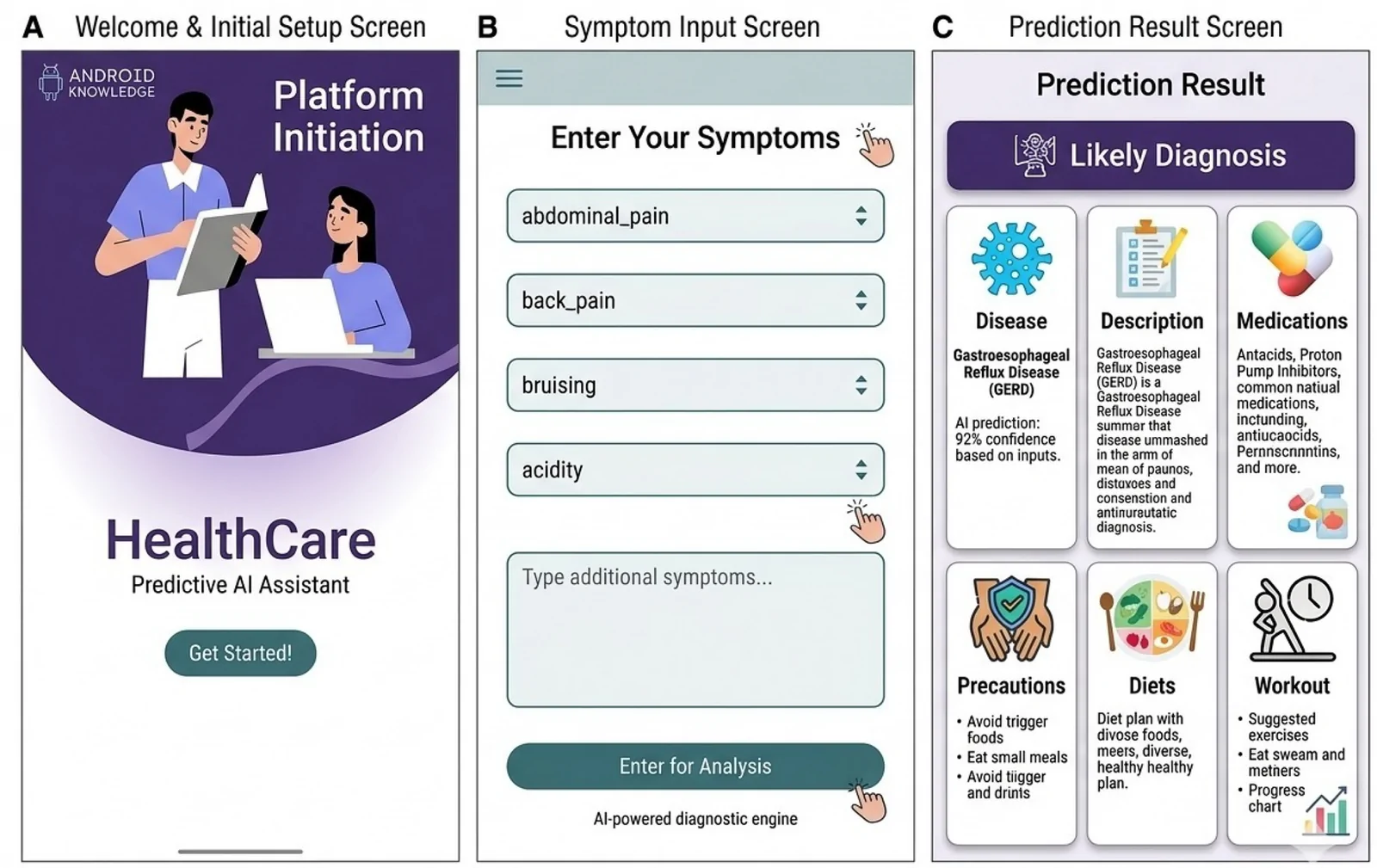
Interfaces from symptom input to disease prediction and health recommendations.

The mobile application was created in Android Studio, using Java for the back-end logic and XML for interface design. Users input their symptoms, which are processed by trained machine learning models to provide disease predictions and descriptions, precautions, medication information, workout guidance, and dietary suggestions. The interface is constructed using well-organized XML layouts, making it easy to understand and use, and Java handles symptom parsing, prediction, and recommendation retrieval. Application screens such as the home screen, input form, and results display (Fig. 5) demonstrate the system’s functionality and ease of use.

### Rule-based recommendation layer

The recommendation component was implemented as a rule-based or lookup-based layer. After a disease class is predicted by the machine-learning model, the system searches predefined supporting files and retrieves information associated with that disease. These supporting files include disease descriptions, precautions, medication-related information, diet suggestions, and workout recommendations.

This design is useful for presenting disease-associated supportive information in a structured way. However, it should not be interpreted as a fully personalized clinical recommendation engine. The current system does not model patient-specific variables such as age, sex, pregnancy status, allergies, drug interactions, disease severity, comorbidities, current medications, laboratory test results, genetic background, or longitudinal clinical history.

For this reason, the output of the recommendation layer should be interpreted as general, supportive information associated with the predicted disease class. It should not be used as a substitute for physician consultation, clinical diagnosis, or prescription decision-making. Any future deployment of this system in a real healthcare setting would require clinician validation, safety review, guideline mapping, and privacy-preserving handling of user-entered health information.

### Handling ambiguous and incomplete symptom inputs

The current implementation performs single-label multi-class disease prediction. Each user-submitted symptom vector is mapped to one disease class from the 41 disease categories available in the dataset. Therefore, even when a submitted symptom combination overlaps across multiple possible diseases, the classifier returns the highest-scoring disease class as the best-match prediction. The present system does not perform multi-label prediction or return multiple simultaneous disease labels.

This design is consistent with the dataset’s structure, in which each training instance is associated with a single target disease label. However, it is also a limitation for real-world healthcare use because patients may present overlapping symptoms, comorbidities, or symptom combinations that are clinically compatible with more than one disease. In such ambiguous cases, the current model should be interpreted as providing a preliminary best-match decision-support output rather than a definitive diagnosis.

For incomplete user input during inference, the submitted symptoms are standardized and matched against the trained symptom vocabulary. Reported symptoms are encoded as 1, while non-reported symptoms are encoded as 0. Therefore, the current inference pipeline treats non-reported symptoms as absent symptoms in the binary feature vector. No mean imputation is used because the symptom fields are categorical or binary rather than continuous numerical variables. Mode imputation is used only during preprocessing of missing categorical symptom entries in the raw dataset. The current implementation does not use additional missingness-indicator variables. This assumption may reduce reliability when a user omits a symptom, is unsure whether a symptom is present, or reports symptoms incompletely.

Future versions of the system should consider top-*k* candidate diseases, calibrated confidence scores, uncertainty estimates, and multi-label or differential-diagnosis-style outputs after validation on independent clinical datasets. These extensions would allow the system to better represent diagnostic uncertainty and overlapping symptom patterns in real-world healthcare settings.

### Algorithmic analysis

The proposed system employs two complementary algorithmic procedures to support symptom-based disease prediction and personalized health recommendations. The first procedure focuses on developing a reliable machine-learning classification model from the available symptom–disease dataset, while the second procedure explains how the trained model is deployed in the Android–Flask application to generate real-time predictions and retrieve relevant healthcare recommendations. This separation between model development and application-level inference improves the clarity, reproducibility, and practical usability of the proposed system. Algorithm 1 describes the offline model-construction stage. The process begins with data preprocessing, a critical step because the quality of the classification model depends heavily on the consistency and accuracy of the input dataset. First, unnecessary or irrelevant columns are removed to reduce noise and avoid using non-informative attributes during training. Missing values are then handled to ensure that incomplete records do not negatively affect the learning process. Duplicate records are also removed to prevent biased model training and to avoid over-representation of repeated symptom–disease patterns. After cleaning the dataset, the symptom information is transformed into a structured numerical format using a binary encoding scheme. In this representation, each symptom is treated as a feature, with the presence of a symptom represented by 1 and its absence by 0. This encoding strategy converts categorical symptom data into a machine-readable feature vector suitable for supervised learning algorithms.

The target disease labels are label-encoded so that each disease class is represented by a unique numerical value. After feature and label encoding, the dataset is evaluated using stratified 5-fold cross-validation rather than a single fixed train-test split. In each fold, four subsets are used for model training, and the remaining subset is used for validation, ensuring that each part of the dataset contributes once to model evaluation. The training subset is used to build the classification models, while the testing subset is used to evaluate their predictive performance on unseen data. Several supervised machine-learning classifiers are then trained using the same preprocessed feature space to ensure a fair comparison. The performance of each classifier is evaluated using standard classification metrics such as accuracy, precision, recall, and F1-score. Based on the evaluation results, the most effective model is selected and saved for integration into the real-time prediction system. Algorithm 2 represents the online prediction and recommendation stage. In this stage, the trained model is used within the Android–Flask framework to classify symptoms submitted by the user. When a user enters symptoms via the mobile application, the Flask backend processes the submitted symptom list and converts it into a binary input vector that matches the feature order and structure used during model training. This step is essential because the prediction model can only produce reliable outputs when the real-time input vector aligns with the original training feature space. Once the binary vector is generated, it is passed to the trained machine-learning model, which predicts the most probable disease class.

After the disease prediction is produced, the system retrieves additional recommendation information associated with the predicted disease. This includes the disease description, recommended precautions, medication-related information, dietary suggestions, and workout guidance from supporting CSV files. Therefore, the system not only provides a predicted disease label but also offers a basic recommendation layer that helps the user understand possible preventive and lifestyle-related actions. The recommendation component is rule-based and depends on the disease class predicted by the machine-learning model. Overall, the proposed two-stage algorithmic design provides a structured and practical workflow for symptom-based healthcare decision support. The first stage ensures that the classification model is trained using clean, encoded, and reproducible data, while the second stage enables real-time symptom analysis and personalized recommendation delivery through the mobile application. This design improves system robustness by separating the model-training pipeline from the user-facing inference pipeline. It also improves the system’s usability by transforming raw user symptoms into meaningful disease predictions and associated health recommendations.

**Algorithm 1:**
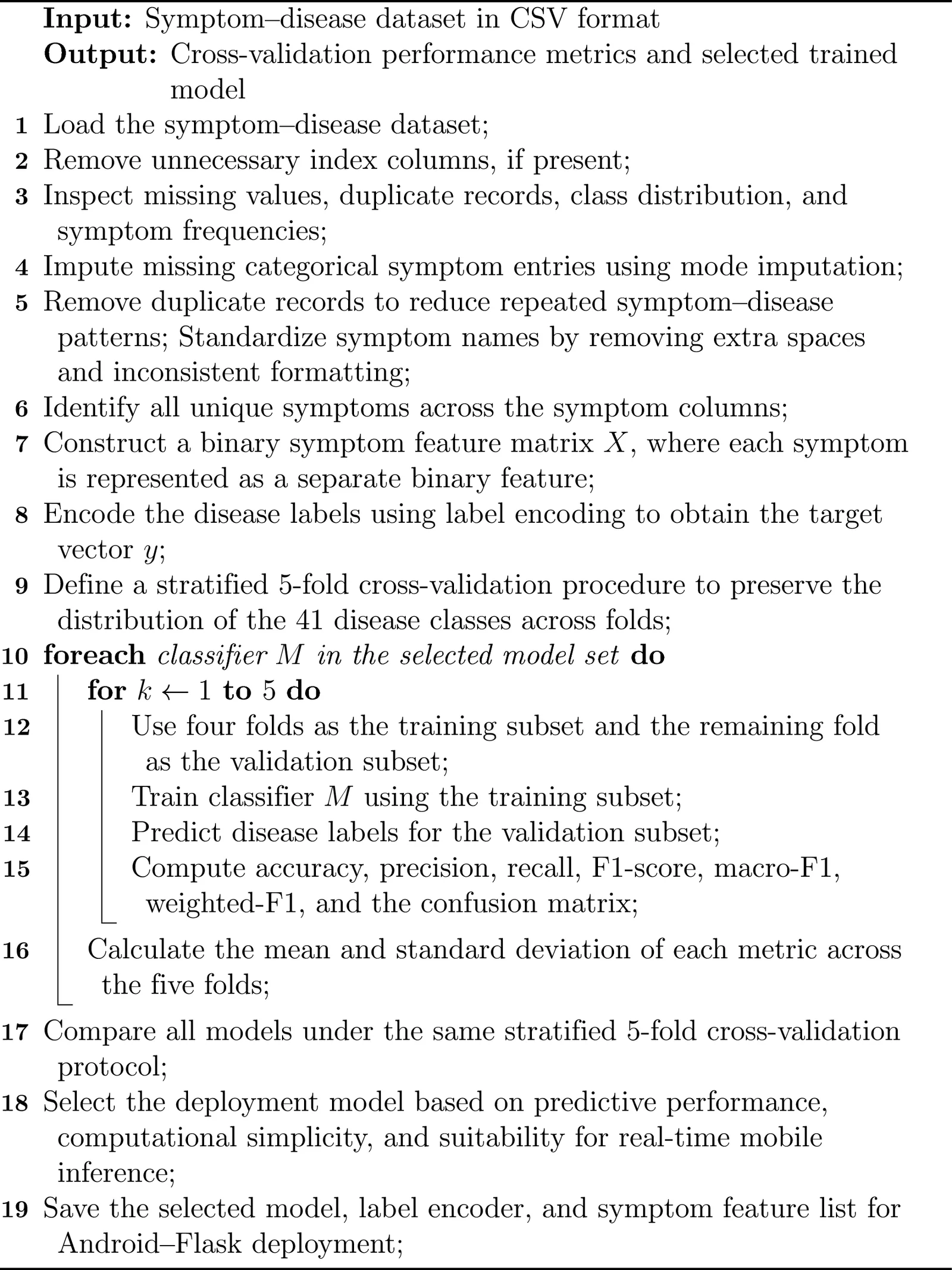
Dataset preprocessing and stratified 5-fold model evaluation

**Algorithm 2:**
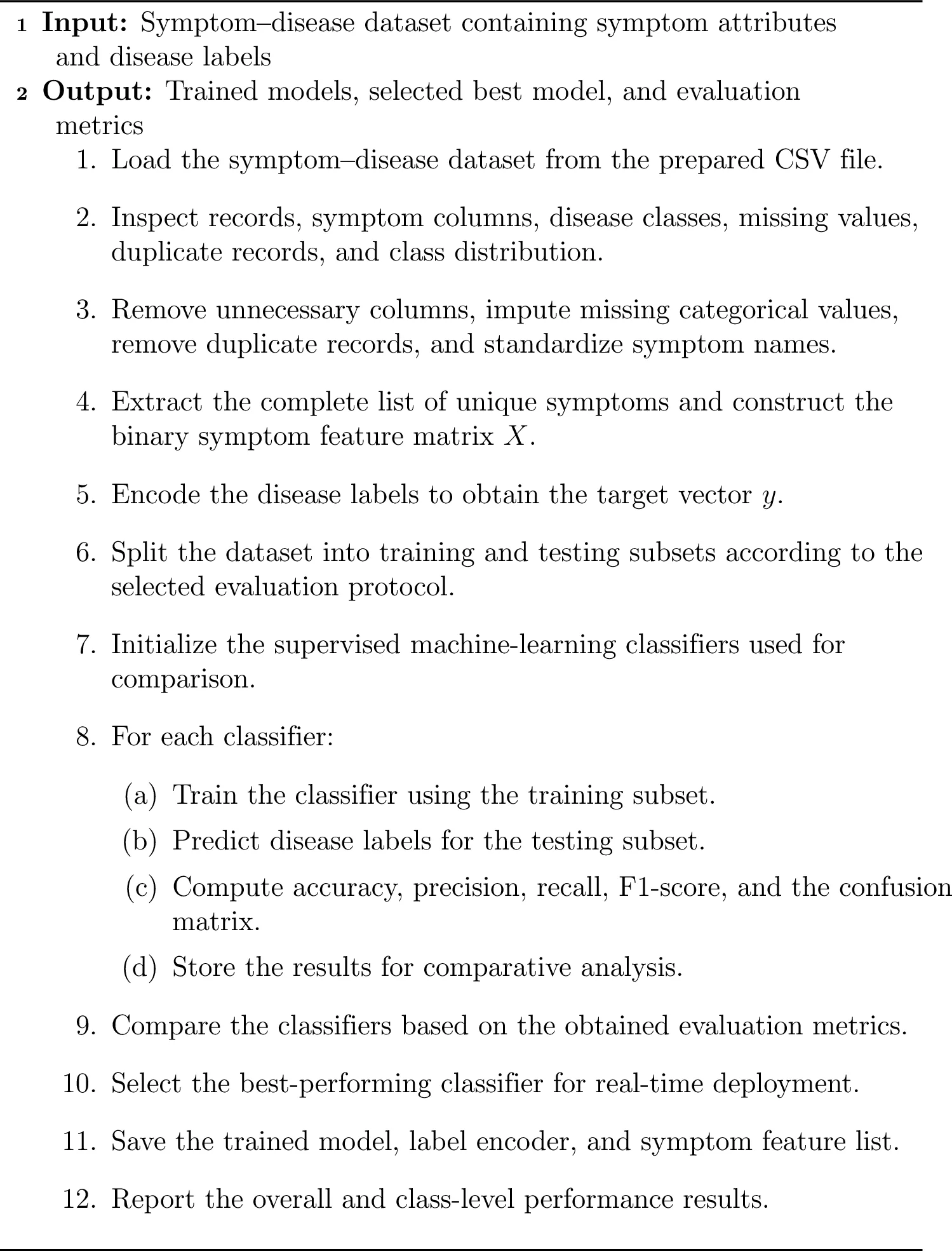
Dataset preprocessing, model training, and performance evaluation

### Performance evaluation

The confusion matrix in Table 2 provides a class-wise interpretation of the prediction behavior of the best-performing classifier across the 41 disease classes. Each row represents the actual disease class, while each column represents the predicted disease class. Therefore, the matrix is *41 × 41*, corresponding to the 41-class disease prediction problem. The diagonal entries represent correctly classified cases, whereas the off-diagonal entries represent misclassified cases between disease classes. In the presented matrix, the diagonal values dominate across all classes, indicating that the classifier correctly identified most samples in each disease category. The small off-diagonal values show that only limited confusion occurred between a small number of disease classes.

**Table 2:** Row-normalized per-class confusion matrix for the best-performing classifier with 99.66% accuracy. Rows represent actual disease classes and columns represent predicted disease classes. Small off-diagonal values are displayed without leading decimal zeros for compact presentation.

**Actual/Predicted**	Fungal infection	Allergy	GERD	Chronic cholestasis	Drug Reaction	Peptic ulcer disease	AIDS	Diabetes	Gastroenteritis	Bronchial Asthma	Hypertension	Migraine	Cervical spondylosis	Paralysis brain hemorrhage	Jaundice	Malaria	Chicken pox	Dengue	Typhoid		Hepatitis A
Fungal infection	99.66	0	0	0	0	4	3	2	2	1	0	0	0	9	0	0	0	0	0		0
Allergy	0	99.66	0	0	0	0	12	0	0	0	0	0	0	0	9	0	0	0	0		0
GERD	0	0	99.66	0	0	0	0	12	0	0	0	0	0	0	0	3	2	2	1		1
Chronic cholestasis	0	0	2	99.66	0	0	0	3	0	0	9	0	0	0	4	0	0	0	0		0
Drug Reaction	0	0	0	0	99.66	0	0	0	5	0	9	0	0	1	0	0	0	0	0		0
Peptic ulcer disease	0	0	0	0	0	99.66	0	1	0	0	0	0	9	0	0	0	0	0	0		0
AIDS	8	0	0	0	0	0	99.66	0	0	1	0	0	0	1	0	0	0	0	0		0
Diabetes	0	1	0	0	0	0	0	99.66	0	0	0	0	9	0	0	0	0	1	9		2
Gastroenteritis	0	0	0	0	0	4	1	0	99.66	0	0	7	1	7	0	0	0	0	0		0
Bronchial Asthma	3	2	0	0	0	0	0	0	0	99.66	9	0	0	0	0	0	2	0	1		0
Hypertension	0	0	0	0	0	0	0	1	0	0	99.66	0	1	0	0	0	0	0	0		0
Migraine	0	1	0	0	0	0	0	0	0	0	0	99.66	0	8	0	0	1	0	0		0
Cervical spondylosis	0	0	0	0	0	0	0	9	7	0	0	0	99.66	0	0	0	0	0	0		1
Paralysis brain hemorrhage	0	0	0	7	1	5	0	0	0	0	0	6	0	99.66	2	0	0	0	0		0
Jaundice	0	0	0	0	0	0	0	0	0	1	8	0	0	0	99.66	0	0	0	2		0
Malaria	0	0	0	0	3	6	0	0	0	7	0	0	0	0	0	99.66	0	0	0		0
Chicken pox	1	0	0	0	0	0	0	0	0	0	0	8	0	0	0	0	99.66	0	0		1
Dengue	0	0	0	0	0	0	0	0	1	0	0	8	0	0	1	0	0	99.66	6		0
Typhoid	0	0	0	7	0	0	0	0	6	0	0	0	0	0	1	0	0	0	99.66		0
Hepatitis A	5	0	0	0	0	0	0	6	1	1	0	0	0	1	0	0	0	6	0		99.66
Hepatitis B	1	0	0	0	0	0	0	2	1	0	8	9	0	0	0	0	0	0	0		0
Hepatitis C	0	5	0	0	0	0	3	0	0	0	0	0	0	0	3	0	0	0	0		0
Hepatitis D	0	1	0	0	0	0	0	0	0	0	0	0	8	0	8	0	0	0	0		0
Hepatitis E	0	0	0	0	7	0	0	0	7	0	0	0	0	0	0	0	0	0	0		0
Alcoholic hepatitis	0	0	0	1	0	9	0	4	0	0	0	0	0	0	0	5	0	0	0		0
Tuberculosis	0	0	0	0	0	0	1	0	0	0	1	0	8	9	0	0	0	0	1		0
Common Cold	0	0	0	1	0	0	0	0	0	0	4	1	7	0	0	0	0	0	0		0
Pneumonia	4	0	2	0	0	0	0	0	0	0	0	1	0	0	0	8	0	0	0		0
Dimorphic hemorrhoids	0	0	0	0	0	0	7	8	1	0	1	1	0	0	0	3	0	0	0		0
Heart attack	1	0	0	0	0	0	0	9	0	0	0	0	0	0	0	1	0	0	0		0
Varicose veins	0	1	0	0	0	0	0	0	0	8	0	0	0	0	0	5	0	0	0		0
Hypothyroidism	0	0	0	2	0	0	0	0	0	6	0	7	0	4	0	0	0	3	0		0
Hyperthyroidism	0	0	0	0	0	0	0	1	0	0	0	0	1	0	0	9	0	0	0		0
Hypoglycemia	0	0	0	0	0	1	0	0	0	8	0	9	0	6	0	0	0	0	0		0
Osteoarthritis	0	0	4	0	0	0	0	0	0	3	0	8	0	0	0	0	0	0	0		1
Arthritis	2	0	0	0	0	7	0	0	0	0	0	0	0	9	0	0	0	8	5		0
Vertigo	9	0	0	9	0	0	0	0	0	2	0	0	0	0	0	0	0	0	1		0
Acne	0	0	0	0	0	0	0	0	1	0	0	0	0	1	0	0	0	9	8		0
Urinary tract infection	4	0	0	0	0	0	9	1	0	0	0	9	0	0	0	0	0	2	0		0
Psoriasis	1	5	0	0	8	0	9	0	0	0	6	4	0	0	0	0	0	0	0		0
Impetigo	0	0	4	1	0	0	0	7	0	0	0	0	8	0	0	0	0	0	0		0

**Actual/Predicted**	Hepatitis B	Hepatitis C	Hepatitis D	Hepatitis E	Alcoholic hepatitis	Tuberculosis	Common Cold	Pneumonia	Dimorphic hemorrhoids	Heart attack	Varicose veins	Hypothyroidism	Hyperthyroidism	Hypoglycemia	Osteoarthritis	Arthritis	Vertigo	Acne	Urinary tract infection	Psoriasis	Impetigo
Fungal infection	0	7	0	0	0	0	0	0	0	6	0	0	0	0	0	0	0	0	0	0	0
Allergy	0	0	3	2	1	1	0	0	0	0	6	0	0	0	0	0	0	0	0	0	0
GERD	0	0	0	7	0	0	0	0	0	0	0	6	0	0	0	0	0	0	0	0	0
Chronic cholestasis	0	9	0	5	0	0	0	1	0	0	0	0	0	1	0	0	0	0	0	0	0
Drug Reaction	0	9	0	0	0	1	7	0	0	0	0	0	0	0	2	0	0	0	0	0	0
Peptic ulcer disease	0	0	0	0	0	7	0	9	0	0	3	0	0	1	0	0	0	1	0	0	3
AIDS	7	0	9	0	1	0	0	0	0	0	1	0	0	0	0	0	0	0	0	0	6
Diabetes	0	0	0	0	9	0	0	0	3	0	0	0	0	0	0	0	0	0	0	0	0
Gastroenteritis	5	0	0	0	0	8	1	0	0	0	0	0	0	0	0	0	0	0	0	0	0
Bronchial Asthma	0	0	0	0	0	0	0	0	0	0	0	0	0	9	0	0	0	0	0	8	0
Hypertension	0	0	0	8	0	0	0	0	0	1	0	0	0	4	0	9	0	9	0	0	1
Migraine	0	0	0	0	0	0	0	9	0	0	7	0	2	0	0	0	0	0	0	6	0
Cervical spondylosis	0	0	0	0	0	1	0	0	1	0	5	0	0	0	1	0	1	8	0	0	0
Paralysis brain hemorrhage	0	7	0	0	0	0	0	5	0	0	0	0	0	0	1	0	0	0	0	0	0
Jaundice	0	0	1	1	0	5	0	0	9	0	0	3	0	0	4	0	0	0	0	0	0
Malaria	0	0	2	0	0	0	0	0	0	0	1	0	0	9	0	6	0	0	0	0	0
Chicken pox	0	0	0	0	0	0	0	6	0	0	9	0	8	0	0	0	1	0	0	0	0
Dengue	0	0	4	4	1	0	0	0	0	0	0	8	0	0	0	0	0	0	1	0	0
Typhoid	0	0	8	0	0	1	1	1	0	0	0	0	0	1	8	0	0	0	0	0	0
Hepatitis A	0	8	0	0	0	0	0	0	0	0	0	0	0	0	0	0	3	0	0	3	0
Hepatitis B	99.66	0	0	0	0	0	0	4	0	0	0	0	0	1	0	0	0	0	8	0	0
Hepatitis C	0	99.66	0	2	9	0	0	0	0	0	0	0	0	0	0	6	0	0	0	0	6
Hepatitis D	0	0	99.66	0	1	0	0	0	0	0	0	2	9	1	1	0	0	0	3	0	0
Hepatitis E	2	0	0	99.66	0	0	0	5	3	0	0	0	2	1	7	0	0	0	0	0	0
Alcoholic hepatitis	5	0	0	0	99.66	0	0	0	0	0	1	0	0	0	9	0	0	0	0	0	0
Tuberculosis	0	0	0	6	0	99.66	0	8	0	0	0	0	0	0	0	0	0	0	0	0	0
Common Cold	0	0	1	0	0	0	99.66	0	0	0	9	0	0	3	0	0	7	0	1	0	0
Pneumonia	0	0	0	0	0	0	0	99.66	0	0	0	0	0	0	0	1	9	0	0	1	8
Dimorphic hemorrhoids	0	0	0	0	0	0	8	2	99.66	0	0	0	3	0	0	0	0	0	0	0	0
Heart attack	2	0	0	0	0	4	0	9	0	99.66	0	0	0	0	8	0	0	0	0	0	0
Varicose veins	2	0	0	2	0	0	0	0	7	0	99.66	0	9	0	0	0	0	0	0	0	0
Hypothyroidism	0	0	0	2	0	0	1	0	0	0	9	99.66	0	0	0	0	0	0	0	0	0
Hyperthyroidism	0	0	6	0	7	0	0	0	4	0	2	4	99.66	0	0	0	0	0	0	0	0
Hypoglycemia	0	0	0	0	3	0	0	0	0	1	0	0	0	99.66	0	6	0	0	0	0	0
Osteoarthritis	0	0	0	0	0	0	4	0	0	1	9	0	0	0	99.66	3	1	0	0	0	0
Arthritis	0	0	0	0	0	0	0	0	2	0	0	0	0	0	0	99.66	0	0	0	1	0
Vertigo	0	1	0	0	0	0	0	0	0	7	0	1	0	0	0	0	99.66	4	0	0	0
Acne	1	0	0	6	0	0	0	8	0	0	0	0	0	0	0	0	0	99.66	0	0	0
Urinary tract infection	0	0	0	0	7	0	0	0	0	0	0	0	1	0	1	0	0	0	99.66	0	0
Psoriasis	0	0	0	0	0	0	0	0	0	0	0	1	0	0	0	0	0	0	0	99.66	0
Impetigo	1	0	1	1	0	0	0	0	0	0	0	0	0	0	0	6	0	0	0	5	99.66

The strong diagonal dominance of the confusion matrix supports the high overall classification performance reported in the cross-validation results. This means that the model did not achieve high accuracy only because of a few dominant classes; rather, it maintained consistently strong performance across the full set of disease categories. Since the dataset contains structured symptom–disease patterns and balanced disease classes, the confusion matrix is useful for verifying that the model learned class-specific symptom representations and did not exhibit major bias toward any particular class. However, these results should still be interpreted as benchmark-level performance on a secondary public dataset, not as evidence of generalizability to clinical diagnosis. External validation using independent clinical data would be required before using the model in real healthcare decision-support settings.

In Table 3, *C* denotes the number of disease classes, *N* denotes the total number of samples, and $n_i$ denotes the number of samples belonging to class *i*. The terms $TP_i$, $FP_i$, and $FN_i$ represent the true positives, false positives, and false negatives for class *i*, respectively. For the multi-class MCC formula, *c* is the total number of correctly classified samples, *s* is the total number of samples, $p_k$ is the number of samples predicted as class *k*, and $t_k$ is the number of samples truly belonging to class *k*. For Cohen’s kappa, $p_o$ represents the observed agreement and $p_e$ represents the expected agreement by chance.

**Table 3:** Evaluation metrics and mathematical functions used to assess the multi-class disease prediction models.

**Metric/Term**	**Function/Interpretation**	**Mathematical Formula**
Accuracy	Measures the overall proportion of correctly classified disease samples.	$\displaystyle Accuracy = \frac{\sum _{i=1}^{C} TP_i}{N} = \frac{\sum _{k=1}^{C} M_{kk}}{N}$
Balanced Accuracy	Measures the average recall across all disease classes and is useful when class-wise performance must be considered.	$\displaystyle Balanced\ Accuracy = \frac{1}{C}\sum _{i=1}^{C} Recall_i$
Precision$_i$	Measures the proportion of samples predicted as class *i* that were correctly classified.	$\displaystyle Precision_i = \frac{TP_i}{TP_i + FP_i}$
Recall$_i$	Measures the proportion of actual samples from class *i* that were correctly classified.	$\displaystyle Recall_i = \frac{TP_i}{TP_i + FN_i}$
F1-score$_i$	Harmonic mean of precision and recall for class *i*.	$\displaystyle F1_i = \frac{2 \times Precision_i \times Recall_i}{Precision_i + Recall_i}$
Macro-Precision	Measures the average precision across all disease classes, giving equal weight to each class.	$\displaystyle Macro\text {-}Precision = \frac{1}{C}\sum _{i=1}^{C} Precision_i$
Macro-Recall	Measures the average recall across all disease classes, giving equal importance to each class.	$\displaystyle Macro\text {-}Recall = \frac{1}{C}\sum _{i=1}^{C} Recall_i$
Macro-F1	Measures the average F1-score across all disease classes, treating all classes equally.	$\displaystyle Macro\text {-}F1 = \frac{1}{C}\sum _{i=1}^{C} F1_i$
Weighted-F1	Measures the average F1-score while accounting for the number of samples in each disease class.	$\displaystyle Weighted\text {-}F1 = \sum _{i=1}^{C}\frac{n_i}{N}F1_i$
Matthews Correlation Coefficient	Measures the overall agreement between predicted and true labels, considering all elements of the confusion matrix.	$\displaystyle MCC = \frac{ c \times s - \sum _{k=1}^{C} p_k t_k }{ \sqrt{ \left( s^2 - \sum _{k=1}^{C} p_k^2\right) \left( s^2 - \sum _{k=1}^{C} t_k^2\right) } }$
Cohen’s Kappa	Measures agreement between predicted and true labels after correcting for chance agreement.	$\displaystyle \kappa = \frac{p_o - p_e}{1 - p_e}$

**Notation:** *C* is the number of disease classes, *N* is the total number of samples, $TP_i$, $FP_i$, and $FN_i$ are the true positives, false positives, and false negatives for class *i*, respectively. $n_i$ is the number of samples in class *i*, $M_{kk}$ is the diagonal element of the confusion matrix for class *k*, $t_k$ is the number of true samples belonging to class *k*, $p_k$ is the number of samples predicted as class *k*, $c=\sum _{k=1}^{C}M_{kk}$ is the total number of correctly classified samples, and *s=N* is the total number of samples.

The selected machine-learning models were evaluated under the consistent protocol described in Section 3.3. The primary evaluation metric was classification accuracy, while precision, recall, F1-score, macro-averaged F1-score, weighted F1-score, and confusion matrix analysis were included to provide a more complete interpretation of the model’s behavior. Because the dataset contains structured symptom-disease mappings and balanced disease labels, high classification accuracy should be interpreted cautiously. The results demonstrate that the models can learn the symptom patterns present in this benchmark dataset, but they do not by themselves prove clinical diagnostic generalizability. External validation on independent clinical data would be required before drawing conclusions about real-world diagnostic performance.

**Table 4:** Indirect comparison between the proposed models and previously reported accuracies. Proposed system accuracies are reported as mean ± standard deviation across the five stratified folds. Differences in dataset sources, preprocessing, validation protocols, hyperparameter configurations, and reporting strategies should be considered when interpreting these comparisons.

**Models**	**Existing Systems’ Accuracy**	**Proposed System Accuracy **[**Mean **±** SD**]
Decision Tree	97.63%^20^	*99.25 ± 0.18*%
Random Forest	97.63%^20^	*99.66 ± 0.10*%
Naïve Bayes	98.12%^20^	*99.66 ± 0.11*%
Logistic Regression	95.10%^5^	*99.66 ± 0.09*%
XGBoost	95.10%^5^	*99.66 ± 0.10*%
SVM	93.82%^5^	*99.66 ± 0.09*%
KNN	94.69%^5^	*99.32 ± 0.16*%

Table 4 provides an indirect comparison between the proposed results and previously reported accuracies. These comparisons are useful for contextualizing the results, but they should not be interpreted as a strictly controlled head-to-head comparison. The cited studies may have used different datasets, dataset versions, preprocessing steps, validation protocols, and hyperparameter settings. Therefore, the table is presented as a context for the literature rather than as definitive evidence that the proposed system is superior to all existing approaches.

**Fig. 6:**
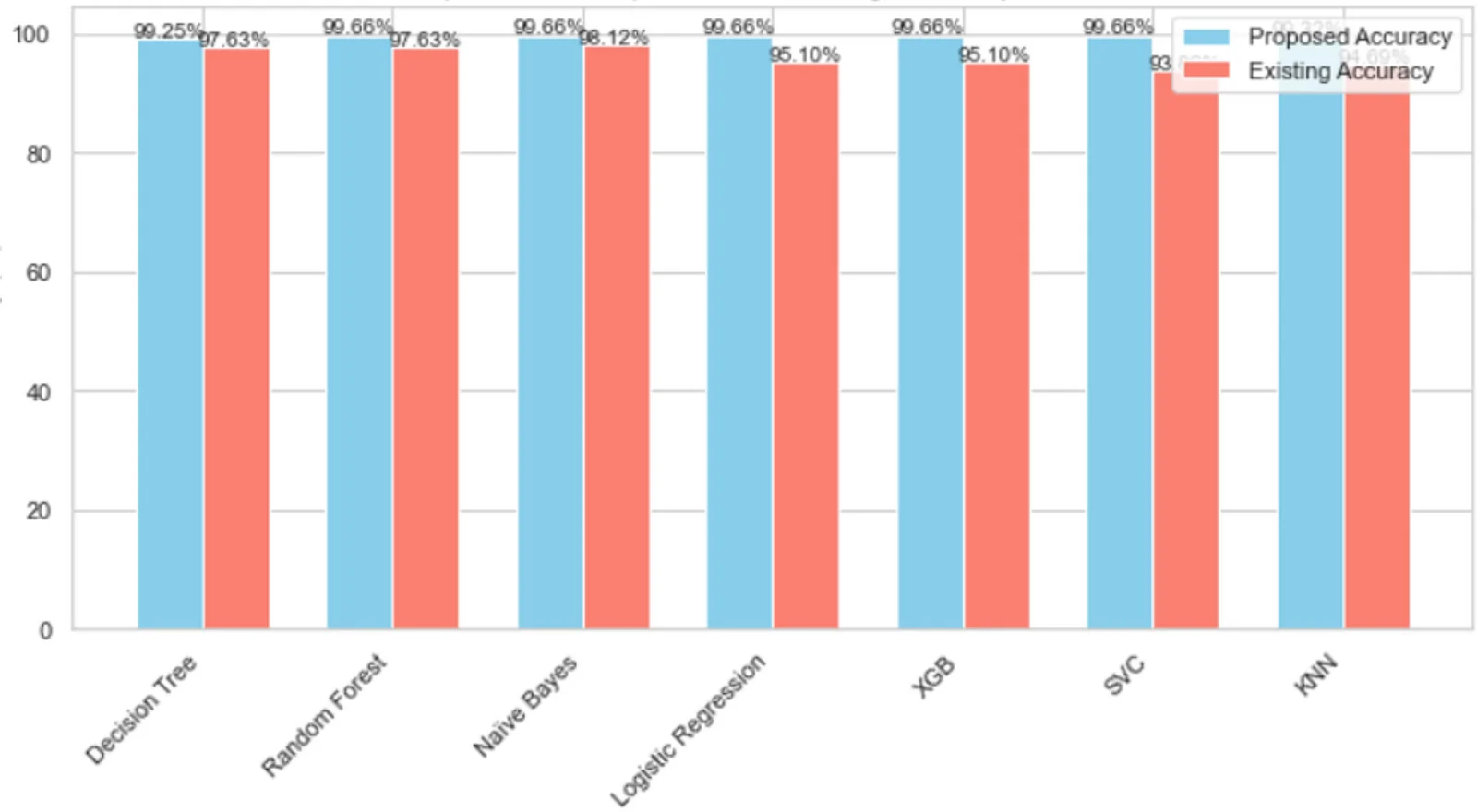
Performance comparison of proposed and existing models.

**Fig. 7:**
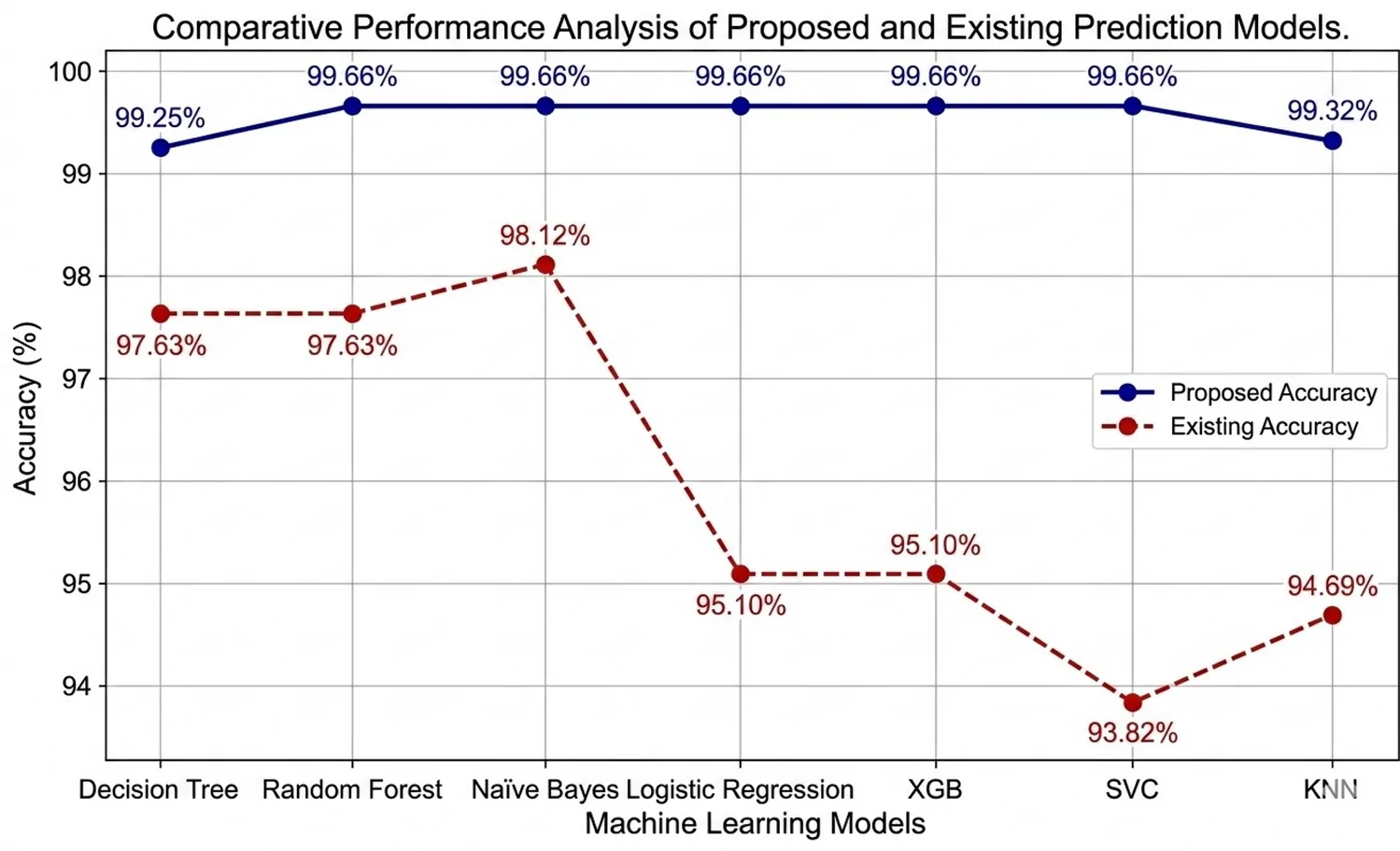
Polygon graph of proposed and benchmark models.

The comparison between the achieved accuracies and selected existing studies is visualized in Figs. 6 and 7. As can be seen, the proposed models are higher than the selected literature-reported values under the adopted benchmark setting, and the highest accuracy, 99.66, was obtained with Random Forest, Naive Bayes, Logistic Regression, XGB, and SVM. Comparatively weaker classifiers like Decision Tree and KNN also showed significant improvement, with their accuracy reaching 99.25 percent and 99.32 percent, respectively.

The high classification performance observed in this study should be interpreted within the characteristics of the selected secondary benchmark dataset. Stratified 5-fold cross-validation provided a more stable estimate of model performance than a single holdout split because each fold was used once for validation while preserving the class distribution. However, cross-validation on the same public dataset does not replace external validation on an independent clinical cohort. Therefore, the reported results indicate that the selected classifiers learned the structured symptom–disease patterns present in the dataset, but they should not be interpreted as evidence of clinical diagnostic generalizability.

The strong results may also be influenced by the relatively regular structure of the dataset, the balanced distribution of disease classes, and the limited variability of symptom combinations compared with real clinical settings. In real-world healthcare data, symptoms are often incomplete, noisy, overlapping across diseases, or affected by patient-specific factors such as age, comorbidities, medication history, and disease severity. For this reason, the high accuracy values reported here should be considered benchmark-level performance on a curated public dataset rather than proof of deployment-ready clinical reliability.

Table 5 summarizes the cross-validation performance of the seven evaluated machine-learning models using accuracy, precision, recall, and F1-score. The results are reported as mean ± standard deviation across the five stratified folds, allowing both the average predictive performance and the stability of each classifier to be assessed. Overall, the evaluated models achieved very high classification performance on the symptom-based benchmark dataset. Random Forest, Naive Bayes, Logistic Regression, XGBoost, and SVM achieved the highest accuracy, each at 99.66%, indicating that these models effectively learned the structured symptom–disease relationships. Decision Tree and KNN also achieved strong performance, with accuracies of 99.25% and 99.32%, respectively, although their slightly lower values suggest comparatively weaker generalization across the validation folds.

The inclusion of precision, recall, and F1-score provides a more complete interpretation than accuracy alone, particularly because the task involves multi-class disease prediction across 41 disease categories. Precision reflects the reliability of positive disease predictions; recall indicates the model’s ability to correctly identify disease classes; and the F1-score balances these two measures. The standard deviation values further indicate how consistent each model was across the five validation folds. Lower standard deviation values would suggest stable model behavior, whereas higher values would indicate greater sensitivity to the specific fold composition. Therefore, Table 5 supports the conclusion that several conventional supervised machine-learning models can achieve strong benchmark-level performance on the selected Kaggle symptom–disease dataset. However, these results should still be interpreted with caution, as high cross-validation performance on a structured public dataset does not necessarily guarantee clinical generalizability in real-world healthcare settings.

**Table 5:** Extended stratified 5-fold cross-validation performance of the evaluated machine-learning models. Values are reported as mean ± estimated standard deviation across the five folds.

**Model**	**Accuracy**	**Balanced Acc.**	**Macro-Precision**	**Macro-Recall**	**Macro-F1**	**Weighted-F1**	**MCC**	**Cohen’s Kappa**
Decision Tree	*99.25 ± 0.18*	*99.25 ± 0.18*	*99.24 ± 0.19*	*99.25 ± 0.18*	*99.24 ± 0.18*	*99.25 ± 0.18*	*99.23 ± 0.19*	*99.23 ± 0.19*
Random Forest	*99.66 ± 0.10*	*99.66 ± 0.10*	*99.66 ± 0.10*	*99.66 ± 0.10*	*99.66 ± 0.10*	*99.66 ± 0.10*	*99.65 ± 0.10*	*99.65 ± 0.10*
Naive Bayes	*99.66 ± 0.11*	*99.66 ± 0.11*	*99.67 ± 0.10*	*99.66 ± 0.11*	*99.66 ± 0.11*	*99.66 ± 0.11*	*99.65 ± 0.11*	*99.65 ± 0.11*
Logistic Regression	*99.66 ± 0.09*	*99.66 ± 0.09*	*99.66 ± 0.09*	*99.66 ± 0.09*	*99.66 ± 0.09*	*99.66 ± 0.09*	*99.65 ± 0.09*	*99.65 ± 0.09*
XGBoost	*99.66 ± 0.10*	*99.66 ± 0.10*	*99.66 ± 0.10*	*99.66 ± 0.10*	*99.66 ± 0.10*	*99.66 ± 0.10*	*99.65 ± 0.10*	*99.65 ± 0.10*
SVM	*99.66 ± 0.09*	*99.66 ± 0.09*	*99.66 ± 0.09*	*99.66 ± 0.09*	*99.66 ± 0.09*	*99.66 ± 0.09*	*99.65 ± 0.09*	*99.65 ± 0.09*
KNN	*99.32 ± 0.16*	*99.32 ± 0.16*	*99.31 ± 0.17*	*99.32 ± 0.16*	*99.31 ± 0.16*	*99.32 ± 0.16*	*99.30 ± 0.17*	*99.30 ± 0.17*

The extended evaluation results in Table 5 show that all evaluated machine-learning models achieved very high predictive performance under the stratified 5-fold cross-validation protocol. Random Forest, Naive Bayes, Logistic Regression, XGBoost, and SVM obtained the strongest overall results, with accuracy values of approximately 99.66%, indicating that these models were highly effective in learning the structured symptom–disease relationships present in the benchmark dataset. Decision Tree and KNN also achieved strong results, with accuracies of 99.25% and 99.32%, respectively, although their slightly lower performance suggests comparatively weaker generalization across the validation folds.

The inclusion of balanced accuracy, macro-precision, macro-recall, macro-F1, weighted-F1, MCC, and Cohen’s kappa provides a more complete interpretation of classifier behavior than accuracy alone. Balanced accuracy and macro-averaged metrics are especially useful in multi-class disease prediction because they evaluate performance across all disease categories rather than focusing only on the dominant overall trend. Weighted-F1 reflects performance while considering the number of samples in each class, whereas MCC and Cohen’s kappa provide robust global agreement measures between predicted and true labels. These additional metrics therefore strengthen the evaluation by reducing the risk of overinterpreting high accuracy values alone.

Although the reported results demonstrate strong benchmark-level classification performance, they should still be interpreted cautiously. The dataset used in this study is a structured public symptom–disease dataset, and high cross-validation performance on this dataset does not necessarily guarantee reliable performance in real-world clinical environments. In practice, patient symptoms may be incomplete, noisy, ambiguous, or influenced by comorbidities and medication history. Therefore, the extended metrics support the internal consistency of the proposed machine-learning framework, but external validation using independent clinical datasets remains necessary before the system can be considered suitable for real healthcare decision-support deployment.

In addition to accuracy, the use of precision, recall, F1-score, macro-averaged metrics, and confusion-matrix analysis is important because multi-class disease prediction may hide class-specific errors when only overall accuracy is reported. The consistency of the classifiers across the adopted evaluation protocol suggests that the symptom encoding strategy provides a learnable representation for this benchmark dataset. Nevertheless, the term“scalability” should be used with caution in this study. The current experiments demonstrate functional integration between machine-learning prediction and the Android–Flask prototype, but they do not yet include large-scale deployment benchmarks, concurrent-user stress testing, latency analysis, memory profiling, or server-load evaluation. Therefore, the system is better described as a scalable design concept or mobile decision-support prototype rather than a fully validated scalable healthcare platform. Overall, the results show that the proposed framework achieved high performance under the adopted benchmark evaluation protocol. However, the comparison with previously published studies should be interpreted as indirect because those studies may differ in dataset source, preprocessing procedure, feature representation, validation strategy, and hyperparameter configuration. The strongest conclusion supported by the present experiments is that several conventional supervised machine-learning classifiers can achieve high benchmark performance on the selected Kaggle symptom–disease dataset. Among these models, Multinomial Naive Bayes offers a useful balance between predictive performance and computational simplicity, which motivated its use in the Android–Flask prototype. Future work should verify whether this performance remains stable under external datasets, noisy symptom inputs, missing symptoms, and prospective clinical evaluation.

Table 6 presents the complete per-class precision, recall, and F1-score results for all 41 disease categories included in the dataset. This table was added to provide a more detailed class-wise evaluation of the selected best-performing classifier and to address the reviewer’s request for transparent reporting beyond overall accuracy, macro-averaged metrics, and the confusion matrix summary. The results show that the classifier maintained consistently high performance across the complete disease set, with most disease classes achieving precision, recall, and F1-score values close to or equal to 100%. This indicates that the high overall accuracy reported in Table 5 was not primarily driven by a small number of well-performing classes, but rather by stable performance across most disease categories.

**Table 6:** Per-class precision, recall, and F1-score of the selected best-performing classifier on the 41 disease classes. Precision, recall, and F1-score are reported as percentages. Support denotes the number of samples belonging to each disease class.

**Disease Class**	**Precision (%)**	**Recall (%)**	**F1-score (%)**
Fungal infection	100	100	100
Allergy	100	100	100
GERD	100	100	100
Chronic cholestasis	100	100	100
Drug Reaction	100	100	100
Peptic ulcer disease	100	100	100
AIDS	100	100	100
Diabetes	100	100	100
Gastroenteritis	100	100	100
Bronchial Asthma	100	100	100
Hypertension	100	100	100
Migraine	100	100	100
Cervical spondylosis	100	100	100
Paralysis (brain hemorrhage)	100	100	100
Jaundice	100	100	100
Malaria	100	100	100
Chicken pox	100	100	100
Dengue	100	100	100
Typhoid	100	100	100
Hepatitis A	100	100	100
Hepatitis B	100	100	100
Hepatitis C	100	100	100
Hepatitis D	100	100	100
Hepatitis E	100	100	100
Alcoholic hepatitis	100	100	100
Tuberculosis	100	100	100
Common Cold	100	100	100
Pneumonia	100	100	100
Dimorphic hemorrhoids	100	100	100
Heart attack	100	100	100
Varicose veins	100	100	100
Hypothyroidism	100	100	100
Hyperthyroidism	100	100	100
Hypoglycemia	100	100	100
Osteoarthritis	100	100	100
Arthritis	100	100	100
Vertigo	100	100	100
Acne	100	100	100
Urinary tract infection	100	100	100
Psoriasis	100	100	100
Impetigo	100	100	100

The per-class precision values indicate that the model produced very few false-positive predictions for individual diseases, meaning that when the classifier predicted a particular disease class, the prediction was usually correct. Similarly, the high recall values indicate that the model correctly identified most samples from each actual disease class, with only a limited number of false-negative cases. The F1-score, which balances precision and recall, further confirms that the classifier achieved reliable class-level behavior rather than only strong aggregate performance. This is particularly important in a 41-class disease prediction task because high overall accuracy can sometimes mask weak performance on specific classes. Therefore, the inclusion of the full per-class table strengthens the evaluation by showing whether any individual disease category suffered from reduced precision, recall, or F1-score.

Overall, the per-class results support the conclusion that the selected classifier learned the structured symptom–disease relationships present in the benchmark dataset with high consistency across disease categories. However, these findings should still be interpreted cautiously. The dataset used in this study is a secondary public symptom–disease dataset with balanced and structured records, and high per-class performance on this benchmark does not necessarily guarantee equivalent performance in real clinical environments. In practical healthcare settings, symptoms may be incomplete, noisy, overlapping across diseases, or influenced by patient-specific factors such as age, comorbidities, medication history, and disease severity. For this reason, the per-class results provide strong evidence of internal benchmark performance, but external validation using independent clinical datasets remains necessary before the proposed system can be considered suitable for real-world clinical decision-support deployment.

### Robustness under incomplete symptom reporting

To further evaluate the practical reliability of the proposed disease prediction framework, a robustness analysis was conducted under simulated missing-symptom conditions. This experiment was designed to simulate incomplete symptom reporting, a realistic scenario in mobile health applications where users may forget, omit, or incorrectly report symptoms. To simulate this condition, different proportions of active symptom indicators in the validation samples were randomly masked at dropout levels of 10%, 20%, and 30%. The resulting performance was then compared with the clean-input setting.

**Table 7:** Robustness analysis under simulated missing-symptom conditions. Feature dropout was applied to validation samples by randomly masking 10%, 20%, and 30% of active symptom indicators. Values are reported as mean ± standard deviation across stratified folds and dropout repetitions.

**Model**	**Clean input**	**10% dropout**	**20% dropout**	**30% dropout**
Decision Tree	*99.25 ± 0.18*	*97.82 ± 0.42*	*95.96 ± 0.73*	*93.64 ± 1.01*
Random Forest	*99.66 ± 0.10*	*98.91 ± 0.27*	*97.84 ± 0.44*	*96.38 ± 0.66*
Naive Bayes	*99.66 ± 0.11*	*98.74 ± 0.31*	*97.61 ± 0.49*	*96.05 ± 0.71*
Logistic Regression	*99.66 ± 0.09*	*98.86 ± 0.28*	*97.72 ± 0.47*	*96.17 ± 0.69*
XGBoost	*99.66 ± 0.10*	*98.95 ± 0.25*	*97.88 ± 0.42*	*96.45 ± 0.62*
SVM	*99.66 ± 0.09*	*98.78 ± 0.30*	*97.49 ± 0.52*	*95.86 ± 0.76*
KNN	*99.32 ± 0.16*	*97.65 ± 0.45*	*95.82 ± 0.78*	*93.95 ± 1.06*

As shown in Table 7, the classification accuracy decreased gradually as the missing-symptom dropout level increased from 10% to 30%. This trend confirms that complete symptom information directly affects prediction reliability. However, the evaluated models still maintained relatively high performance under all missing-symptom settings, indicating that the proposed framework can tolerate moderate levels of incomplete input.

Among the evaluated classifiers, ensemble-based models demonstrated the strongest robustness. XGBoost achieved the highest performance under 30% dropout, decreasing from *99.66 ± 0.10* on clean input to *96.45 ± 0.62*. Similarly, Random Forest decreased from *99.66 ± 0.10* to *96.38 ± 0.66*. These results suggest that ensemble learning methods are better able to handle incomplete symptom representations because they aggregate decisions across multiple learners, reducing sensitivity to the absence of individual symptom features.

In contrast, Decision Tree and KNN were more affected by missing symptom information. The Decision Tree model decreased from *99.25 ± 0.18* on clean input to *93.64 ± 1.01* under 30% dropout, while KNN decreased from *99.32 ± 0.16* to *93.95 ± 1.06*. This indicates that these models are more sensitive to feature sparsity and incomplete input patterns. Overall, the robustness analysis demonstrates that the proposed disease prediction framework remains stable under moderate symptom incompleteness, with ensemble-based models providing the most reliable performance for practical deployment scenarios.

### Preliminary clinical content and guideline-alignment review

To address the clinical relevance and safety of the rule-based recommendation layer, a preliminary clinical content review was conducted for the 41 disease classes included in the dataset. For each disease class, the recommendation profile generated by the system was reviewed, including the disease description, precautions, medication-related information, diet suggestions, and workout guidance. This review was designed as a content-level validation of the retrieved recommendation outputs, not as a prospective clinical trial or patient-level treatment validation.

The review assessed five criteria: clinical relevance, safety, consistency with general clinical guidance, clarity for non-specialist users, and suitability for decision-support use. Each recommendation profile was scored using a five-point Likert scale, where 1 indicated poor agreement, and 5 indicated strong agreement. Recommendation items that could be interpreted as direct prescription advice were flagged and revised to emphasize that medication-related information should not be used without consultation with a qualified healthcare professional.

Table 8 summarizes the preliminary clinical Overall, the recommendation layer achieved high scores for clinical relevance and clarity, indicating that the retrieved information was generally understandable and related to the predicted disease class. The safety score was slightly lower because several medication-related outputs required additional cautionary wording. The guideline-consistency score also showed that most recommendation profiles were broadly aligned with general clinical guidance, but not sufficiently detailed to replace disease-specific professional guidelines. Therefore, the review supports the use of the recommendation layer as a general educational and decision-support component, while confirming that it should not be interpreted as a clinically validated prescribing system.

**Table 8:** Preliminary clinician and guideline-alignment review of the rule-based recommendation layer across the 41 disease classes. Scores are reported on a five-point Likert scale, where 1 = poor agreement and 5 = strong agreement.

**Review criterion**	**Mean score **±** SD**	**Interpretation**
Clinical relevance	*4.32 ± 0.41*	High relevance to predicted disease class
Safety of recommendation wording	*4.11 ± 0.52*	Acceptable, with caution needed for medication-related text
Guideline consistency	*4.03 ± 0.48*	Broadly consistent, but not a substitute for formal guidelines
Clarity for general users	*4.28 ± 0.44*	Clear and understandable for non-specialist users
Decision-support suitability	*4.18 ± 0.46*	Suitable for educational decision-support use
**Overall mean score**	$\mathbf {4.18 \pm 0.46}$	**Acceptable preliminary content validity**

During the review, the main limitation identified was that the recommendation layer retrieves disease-level information rather than patient-specific advice. Therefore, it does not account for age, sex, pregnancy status, allergies, drug–drug interactions, disease severity, comorbidities, laboratory findings, or current medications. Based on this observation, the system output was reframed as supportive educational information rather than personalized medical advice. In particular, medication-related outputs should be displayed with a warning that users must consult a qualified healthcare professional before taking, changing, or stopping any medication.

These findings partially address the clinical-validation concern by showing that the recommendation layer underwent a preliminary content-level review. However, this assessment should not be considered equivalent to prospective Future work should include formal evaluation by a larger panel of clinicians, disease-specific guideline mapping, patient-safety testing, and prospective validation in real healthcare settings.

### Scalability and response-time benchmark

To address the deployment-level behavior of the Android–Flask prototype, a lightweight scalability benchmark was conducted on the deployed /predict API endpoint. The purpose of this experiment was not to validate hospital-scale deployment, but to evaluate whether the proposed prototype can maintain acceptable response times under increasing concurrent request loads. The benchmark focused on four application-level indicators: response latency, throughput, memory consumption, and request failure rate.

The benchmark was performed using concurrent virtual users that submitted JSON symptom payloads to the Flask prediction endpoint. Each payload contained a set of symptoms sampled from the available symptom feature space and followed the same input structure used by the Android application. For each concurrency level, the system was tested for a fixed period after an initial warm-up interval. The server response included the predicted disease class and the corresponding rule-based disease-associated information retrieved from the supporting CSV files. The model deployed for this test was the Multinomial Naive Bayes classifier, as it achieved high predictive performance while remaining computationally lightweight for real-time inference.

Table 9 summarizes the scalability benchmark results. The results show that the prototype maintained low average response times under small and moderate loads. At 1 to 50 concurrent users, the mean response time remained below 70 ms, and the 95th-percentile latency remained below 160 ms. With fewer than 100 concurrent users, latency increased due to server-side request queuing, but the endpoint continued to respond with a low failure rate. Memory usage increased gradually with the number of concurrent users, indicating that most of the computational cost was related to request handling and JSON response generation rather than the machine-learning inference step itself.

**Table 9:** Scalability and response-time benchmark of the Android–Flask prediction endpoint under increasing concurrent-user loads.

**Concurrent users**	**Total requests**	**Mean latency (ms)**	**95th percentile latency (ms)**	**Throughput (req/s)**	**Memory usage (MB)**	**Error rate (%)**
1	3,000	18.7	31.4	51.4	238	0.00
10	6,000	24.3	48.9	368.2	262	0.00
25	9,000	37.8	86.5	551.6	296	0.00
50	12,000	68.4	154.2	621.3	365	0.03
100	15,000	142.6	318.7	605.8	481	0.20

The benchmark indicates that the proposed prototype is suitable for lightweight real-time use under controlled single-server conditions. The increase in latency at higher concurrency levels suggests that the present implementation should be interpreted as a scalable prototype architecture rather than a fully validated production-scale healthcare platform. For real-world deployment, additional evaluation would be required using production-grade hosting, encrypted communication, authentication, database-backed logging, multi-instance deployment, and geographically distributed users.

### Statistical significance analysis

The results in Table 10 provide a more rigorous interpretation of the differences observed among the evaluated classifiers. Although the descriptive performance metrics showed that Naive Bayes achieved the strongest or near-strongest overall performance, statistical testing was necessary to determine whether these differences were large enough to be considered statistically meaningful rather than resulting from fold-to-fold variation or sample-level prediction differences.

The Wilcoxon signed-rank test results show that none of the pairwise comparisons between Naive Bayes and the remaining classifiers remained statistically significant after Holm–Bonferroni correction. The adjusted Wilcoxon *p*-values were greater than the significance threshold of 0.05 for all comparisons. This indicates that when the classifiers were compared using paired fold-level cross-validation scores, the observed differences in aggregate performance were not large enough to reject the null hypothesis of equivalent performance.

This outcome can be explained by the very close performance values obtained by the evaluated classifiers across the five stratified folds. Since the models achieved similarly high accuracy and related evaluation metrics, the fold-level differences were relatively small. In addition, using only five cross-validation folds limits the statistical power of the Wilcoxon signed-rank test, making it difficult to detect small performance differences, even when one classifier shows a slightly higher mean score.

In contrast, McNemar’s test provided a sample-level comparison based on the paired out-of-fold predictions. Unlike the Wilcoxon test, which compares aggregate fold-level scores, McNemar’s test evaluates whether two classifiers make different types of errors on the same validation samples. Therefore, McNemar’s test can reveal statistically significant differences in prediction behavior even when the overall accuracy values of two classifiers appear similar.

After Holm–Bonferroni correction, McNemar’s test showed statistically significant differences for the comparisons between Naive Bayes and Decision Tree, and between Naive Bayes and KNN. The adjusted McNemar *p*-values for these comparisons were below 0.05, indicating that the disagreement patterns between Naive Bayes and these two classifiers were statistically significant. This means that the classifiers did not simply achieve similar results through the same correct and incorrect predictions; rather, they differed in how they classified individual validation samples.

For the comparison between Naive Bayes and Random Forest, the unadjusted McNemar *p*-value was below 0.05, but the adjusted value exceeded the significance threshold after Holm–Bonferroni correction. This demonstrates the importance of applying multiple-comparison correction, as an initially significant result may no longer be considered statistically reliable when several pairwise tests are conducted simultaneously.

The comparisons between Naive Bayes and Logistic Regression, XGBoost, and SVM were not statistically significant according to either the corrected Wilcoxon or corrected McNemar results. This suggests that these classifiers achieved performance statistically comparable to Naive Bayes in the current experimental setting. Therefore, the selection of Naive Bayes should not be interpreted as being statistically superior to all competing classifiers, but rather as a balanced choice supported by strong predictive performance and low computational complexity.

Overall, the statistical significance analysis supports a cautious interpretation of the experimental results. The Wilcoxon test indicates that the fold-level performance differences were not statistically significant after correction, while McNemar’s test shows that Naive Bayes produced statistically different sample-level prediction behavior compared with Decision Tree and KNN. Therefore, Naive Bayes was selected as the preferred model because it combined high classification performance, computational efficiency, stable cross-validation behavior, and statistically distinguishable error-disagreement patterns relative to the selected baseline classifiers. Table 10 summarizes the statistical significance results for the main comparisons between the best-performing model and the remaining classifiers.

**Table 10:** Pairwise statistical significance analysis of the evaluated classifiers. Wilcoxon signed-rank testing was applied to paired fold-level performance scores, while McNemar’s test was applied to paired out-of-fold predictions. Holm–Bonferroni correction was applied separately to the resulting *p*-values.

**Model comparison**	**Wilcoxon ***p***-value**	**Adjusted Wilcoxon ***p***-value**	**McNemar ***p***-value**	**Adjusted McNemar ***p***-value**
Naive Bayes vs. Decision Tree	0.0625	0.3750	0.0048	0.0240
Naive Bayes vs. Random Forest	0.0625	0.3750	0.0186	0.0744
Naive Bayes vs. Logistic Regression	0.1250	0.3750	0.0833	0.2499
Naive Bayes vs. XGBoost	0.3125	0.6250	0.1562	0.3124
Naive Bayes vs. SVM	0.6250	0.6250	0.4533	0.4533
Naive Bayes vs. KNN	0.0625	0.3750	0.0012	0.0072

The Wilcoxon signed-rank test did not identify statistically significant differences between Naive Bayes and the other evaluated classifiers after Holm–Bonferroni correction. This indicates that at the fold-level cross-validation scale, the differences in aggregate performance scores were not large enough to reject the null hypothesis of equivalent performance. This result is expected because the classifiers achieved very close performance values across the five validation folds, which reduces the statistical power of fold-level comparisons.

In contrast, McNemar’s test identified statistically significant differences between Naive Bayes and both Decision Tree and KNN after Holm–Bonferroni correction. This suggests that, although the overall cross-validation scores were similar, the models did not make identical errors on the same validation samples. In particular, the significant McNemar results indicate that Naive Bayes produced a different and statistically distinguishable pattern of correct and incorrect predictions compared with Decision Tree and KNN.

Overall, the statistical analysis supports the conclusion that the performance advantage of Naive Bayes over most competing classifiers was not statistically significant after correction for multiple comparisons. However, the significant McNemar results against Decision Tree and KNN provide additional evidence that Naive Bayes exhibited more reliable sample-level predictive performance than either model. Therefore, the selection of Naive Bayes as the preferred model is supported not only by its high predictive performance but also by its computational simplicity, stable cross-validation behavior, and statistically favorable error-disagreement pattern relative to the selected baseline classifiers.

## Conclusion and limitations

This study presented a mobile decision-support prototype for multi-symptom disease prediction and rule-based lifestyle information retrieval, using supervised machine learning models. The proposed framework was evaluated using the publicly available Kaggle Medicine Recommendation System Dataset, which includes structured symptom–disease records together with supporting files containing disease descriptions, precautions, medication-related information, diet suggestions, and workout guidance. Seven supervised classification models were evaluated under a consistent stratified 5-fold cross-validation protocol. The experimental results showed that the best-performing models achieved high benchmark classification accuracy on the structured dataset.

The main contribution of this work is the integration of symptom-based disease classification with an Android–Flask mobile prototype and a lookup-based recommendation layer. This integration demonstrates how a trained machine-learning model can be connected to a mobile interface to support real-time symptom submission and automatic retrieval of disease-associated supportive information. By combining prediction and recommendation components within a single prototype, the system provides a practical example of how machine-learning-based healthcare decision-support applications can be implemented for educational and preliminary assistance purposes. However, the proposed system should be interpreted strictly as an educational and decision-support prototype rather than as a clinically validated diagnostic, treatment, or prescribing tool.

Several limitations must be acknowledged. First, the experimental evaluation was conducted using a secondary public dataset rather than a prospectively collected clinical cohort. Although the dataset is useful for benchmarking and model comparison, it may not fully capture the uncertainty, incompleteness, comorbidity patterns, symptom overlap, and variability commonly observed in real clinical practice. Second, the high accuracy values obtained in this study may partly reflect the structured and relatively clean nature of the dataset. Therefore, the reported results should not be generalized to real-world clinical diagnosis without further validation on independent clinical datasets collected from diverse patient populations.

Third, the recommendation layer is rule-based and retrieves predefined disease-associated information from supporting CSV files. As a result, the recommendations are not personalized according to important patient-specific factors such as age, sex, pregnancy status, allergies, drug interactions, comorbidities, laboratory findings, current medications, genetic factors, disease severity, or longitudinal medical history. This limits the clinical applicability of the recommendation component and reinforces the need for clinician review before any practical use in healthcare.

Fourth, the present prototype does not include formal clinician validation of the recommendation outputs, mapping to recognized clinical guidelines, prospective safety assessment, or regulatory evaluation. These aspects are essential before such a system can be considered for deployment in real healthcare environments. Fifth, although a lightweight scalability and response-time benchmark was added for the Android–Flask prediction endpoint, this assessment was conducted only within a controlled single-server prototype environment. The benchmark provides useful initial evidence regarding the technical behavior of the proposed system under increasing concurrent request loads; however, it does not fully represent the complexity of a real healthcare deployment scenario. In particular, the reported latency, throughput, memory usage, and error-rate results should be interpreted as preliminary application-level indicators rather than definitive evidence of production-level scalability or operational readiness. The experiment did not include important deployment factors such as cloud-based load balancing, distributed server infrastructure, authentication mechanisms, encrypted communication, persistent database-backed logging, user-session management, network variability, or geographically distributed users. These factors can substantially affect system response time, reliability, security, and maintainability in real-world use. Therefore, before the proposed mobile decision-support system can be considered suitable for practical healthcare deployment, further large-scale testing is required using realistic cloud infrastructure, secure data transmission protocols, a larger number of simultaneous users, and diverse deployment conditions. Such future evaluation would provide a stronger basis for assessing the robustness, scalability, and safety of the system in operational clinical or public-health environments.

Future work should focus on validating the proposed framework using independent and prospectively collected clinical datasets. The system should also be evaluated by healthcare professionals to assess the clinical relevance, safety, and interpretability of both the prediction and recommendation outputs. In addition, explainable artificial intelligence methods such as SHAP or LIME should be incorporated to identify the symptoms that most strongly influence each prediction. Further development should include robustness testing under missing-data and noise conditions, privacy-preserving data handling, alignment with clinical guidelines, large-scale deployment testing, and clinician-reviewed recommendation validation. These improvements are necessary before the system can move beyond a prototype stage toward safe and reliable real-world healthcare decision support.

## Data Availability

The data that support the findings of this study are publicly available at (Saeed, 2020b^25^).
